# Quantifying the biases in metagenome mining for realistic assessment of microbial ecology of naturally fermented foods

**DOI:** 10.1038/srep34155

**Published:** 2016-09-27

**Authors:** Santosh Keisam, Wahengbam Romi, Giasuddin Ahmed, Kumaraswamy Jeyaram

**Affiliations:** 1 Microbial Resources Division, Institute of Bioresources and Sustainable Development (IBSD), Takyelpat Institutional Area, Imphal 795 001, Manipur, India; 2 Department of Biotechnology, Gauhati University, Guwahati 781 014, Assam, India

## Abstract

Cultivation-independent investigation of microbial ecology is biased by the DNA extraction methods used. We aimed to quantify those biases by comparative analysis of the metagenome mined from four diverse naturally fermented foods (bamboo shoot, milk, fish, soybean) using eight different DNA extraction methods with different cell lysis principles. Our findings revealed that the enzymatic lysis yielded higher eubacterial and yeast metagenomic DNA from the food matrices compared to the widely used chemical and mechanical lysis principles. Further analysis of the bacterial community structure by Illumina MiSeq amplicon sequencing revealed a high recovery of lactic acid bacteria by the enzymatic lysis in all food types. However, *Bacillaceae*, *Acetobacteraceae*, *Clostridiaceae* and *Proteobacteria* were more abundantly recovered when mechanical and chemical lysis principles were applied. The biases generated due to the differential recovery of operational taxonomic units (OTUs) by different DNA extraction methods including DNA and PCR amplicons mix from different methods have been quantitatively demonstrated here. The different methods shared only 29.9–52.0% of the total OTUs recovered. Although similar comparative research has been performed on other ecological niches, this is the first in-depth investigation of quantifying the biases in metagenome mining from naturally fermented foods.

Cultivation-independent metagenomic analyzes are increasingly used to understand the microbial ecology of natural food fermentation^1^,^2^. The advances in next-generation sequencing (NGS) techniques and cheaper sequencing cost^3^ fuelled this metagenomic studies, which led to unprecedented insights into the complex microbial ecology of diverse fermented foods^4^,^5^,^6^. Among the available NGS platforms, Illumina MiSeq sequencing with paired-end read of 2 × 300 bp is adequate for barcoded amplicon sequencing of rRNA gene-based metagenomic studies^7^,^8^. However, cultivation-independent rRNA gene-based microbial ecology studies are associated with systemic biases that are related to the choice of DNA extraction methods, variable region of rRNA gene targeted, selection of primers and the molecular analysis platform used^9^. A recent analysis of the metadata of human gut microbiota showed that the microbial communities clustered by studies, indicating that experimental protocol plays a major role in shaping the results^9^. Although universal primers and sequencing pipeline can be uniformly applied, DNA extraction procedures will vary depending on the kind of samples analyzed, particularly for fermented foods where there is a vast difference in the physical and chemical nature of the raw materials used in the fermentation. Depending on its nature, some food matrices may require pre-treatment steps before DNA extraction^1^.

The use of standardized DNA extraction protocol is feasible in large-scale sequencing projects like the Human Microbiome Project and the Earth Microbiome Project where the samples are relatively homogenous. However, the sheer diversity and complexity of the raw materials used in preparing different fermented foods make it challenging, if not impractical, to use a uniform DNA extraction protocol in all cases. Up to a certain extent, commercial extraction kits have mitigated this problem by providing a simple and quick way to extract DNA. Nevertheless, such kits based on chemical or mechanical lysis principles are available only for common food matrices and cannot be readily applied to a novel, uncharacterized and complex food like fermented bamboo shoot products. Moreover, studies comparing the efficiency of kits with in-house developed methods suggest that the performance of different kits are variable and compared poorly with the other methods^10^,^11^,^12^. Hence, optimization of DNA extraction method becomes necessary for accurate and realistic microbial ecology studies. It is also equally important in microbial diagnostics to recover and detect low abundant pathogens from the complex microbial community^13^.

Metagenomic DNA is generally extracted in two ways, either by extracting the microbial cells from the food matrix followed by subsequent lysis or direct *in situ* lysis^14^,^15^. The most commonly used approach involves the *in situ* lysis of cells by using different lytic agents like enzymes^16^, chemicals^12^, mechanical agents^17^,^18^, sonication^14^ or a combination of these different principles^16^,^19^,^20^,^21^. However, different lysis principles are biased towards certain taxa as all microbial groups do not have the same sensitivity to different lytic agents owing to differences in their cell wall structure and composition^4^. For example, Gram positive bacteria are better suited to harsh lysis mechanisms^22^ but these may cause degradation of the nucleic acids in the suspension. Hence, it is critical that the extraction methods should have similar lysis efficiency over all taxa present in the food matrix so that a fair representation of the true microbial community can be depicted^23^. Moreover, the dominant bacterial phylum *Firmicutes* present in fermented foods is widely recognised as tough to get lysed.

We used eight different DNA extraction methods with one or more combination of cell lysis principles (chemical, mechanical, enzymatic, thermal and sonication) to evaluate the impact of DNA extraction procedures on the assessment of the microbial ecology of four different types of naturally fermented foods (bamboo shoot, milk, fish and soybean). To quantify the biases due to DNA extraction methods, we applied different molecular approaches including Illumina MiSeq amplicon sequencing for assessing eubacterial and yeast communities. This is the first study which employs a combination of cultivation-independent techniques like PCR- denaturing gradient gel electrophoresis (DGGE), qPCR and Illumina sequencing to examine the impact of DNA extraction method on the elucidation of microbial community ecology of four diverse naturally fermented food types. The findings from this work will enable the food microbiologists to study the cultivation-independent microbial ecology of fermented foods with minimum biases.

## Results

### Different extraction methods recovered different yield of metagenomic DNA

**Figure 1:**
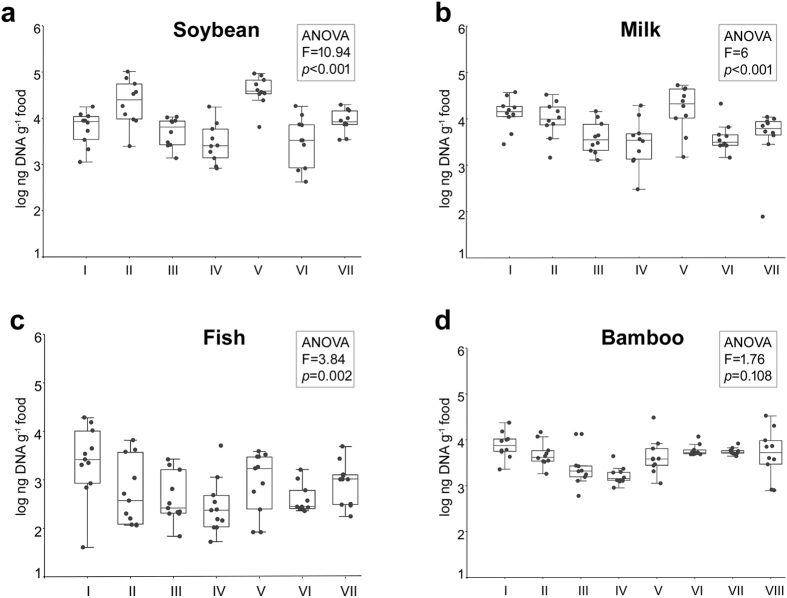
Variation in the metagenomic DNA yield of different extraction methods (I–VIII) as observed in fermented (**a**) soybean (**b**) milk (**c**) fish and (**d**) bamboo shoot. Each method represents data from ten independent replicates. ANOVA highlights the overall significant difference in the DNA yield between extraction methods for each food type. The *p* value for pairwise comparison of the extraction methods in DNA yield is listed in Supplementary Table S1.

The differences in the metagenomic DNA recovery from varied types of fermented foods by eight different extraction methods are shown in Fig. 1. Higher DNA recovery in fermented soybean (4.5–47.8 μg.g^−1^) and milk products (5.28–24.49 μg.g^−1^) compared to fermented bamboo shoot (1.83–9.28 μg.g^−1^) and fish products (0.5–6.18 μg.g^−1^) was observed. In general, the enzymatic methods (II, V) recovered maximum DNA yield from fermented soybean and milk products whereas the non-enzymatic method-I recovered maximum DNA from the fermented bamboo shoot and fish products. Across the food types, the in-house developed methods (III, IV) and the commercial method (VI) showed poor DNA recovery. The DNA recovered by all the methods were of good quality (A_260/280_ = 1.7–2.3) with no PCR inhibitors. For each food type, the efficiency of DNA recovery strongly depended on the extraction method applied and a single method cannot efficiently extract DNA from all the food types.

### Recovery of microbial communities based on the DNA extraction principles

**Figure 2:**
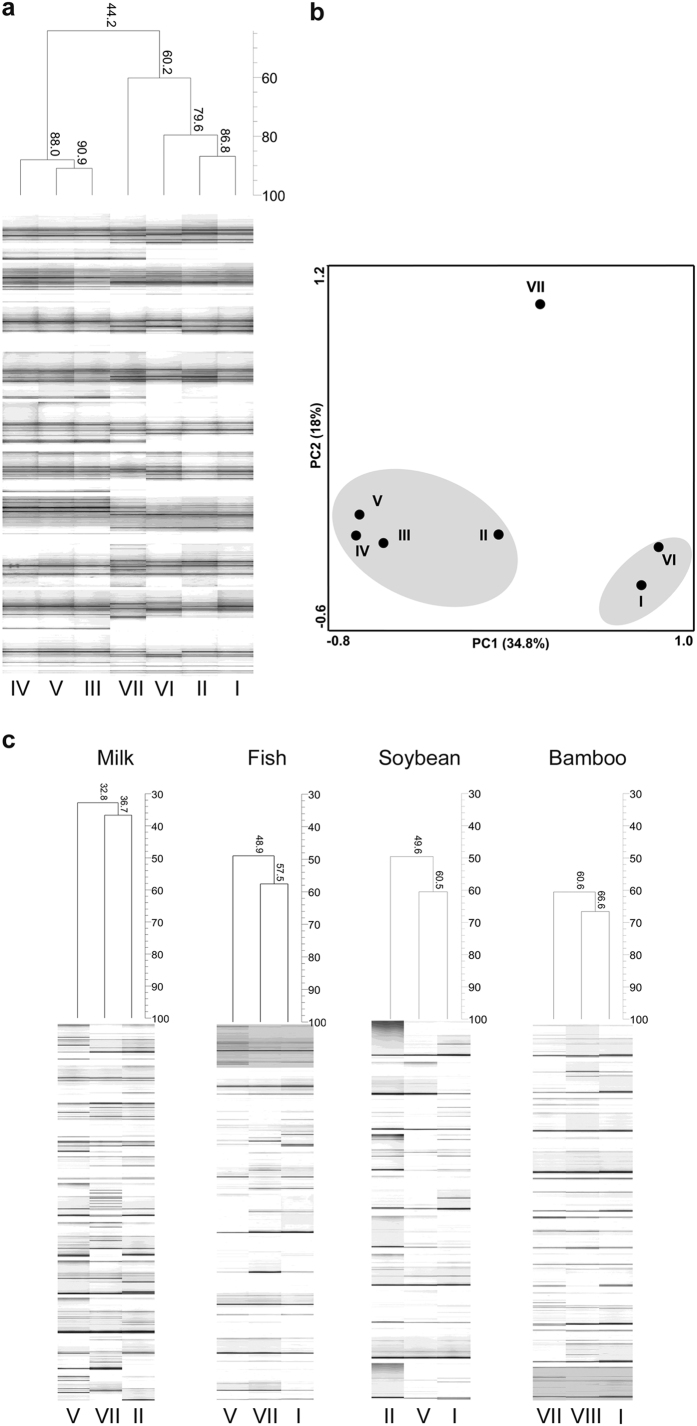
PCR-DGGE fingerprinting of eubacterial and yeast communities showed that DNA extraction methods with similar lysis principles clustered together. (**a**) Pearson correlation based UPGMA clustering of normalized eubacterial community PCR-DGGE fingerprints obtained from fermented milk (n = 10) showing clustering of different extraction methods. The analysis was performed in GelCompar II v6.5 with band matching performed at 1% position tolerance. Value at the nodes represents distance similarity. (**b**) PCA plotting of the methods using normalized PCR-DGGE fingerprints of eubacterial communities generated from all food types (n = 10 each) revealed the clustering of methods based on cell lysis principles. Clustering of the methods based on enzymatic lysis principle (II, III, IV, V) and non-enzymatic lysis principles (I, VI) is highlighted. (**c**) Dendrogram based on yeast community PCR-DGGE fingerprints obtained using the selected three different extraction methods in fermented milk, fish, soybean and bamboo shoot (n = 10 each). Text related to PCR-DGGE optimization is included in Supplementary Note.

PCR-DGGE analysis of both the eubacterial and yeast communities were performed to understand the impact of extraction methods on the assessment of microbial community structure and diversity, as well as to verify whether the variation in DNA yield among the methods influenced the microbial community recovery. Pearson correlation based UPGMA clustering of the eubacterial PCR-DGGE profiles showed that the extraction methods with similar lysis principles mostly clustered together. This impact was clearly visible in fermented milk products in which the two major groups were clustered at 44% similarity (Fig. 2a). For a better comparison of the impact of different methods on the recovery of microbial community structure, an unsupervised principal component analysis (PCA) plotting of the extraction methods using normalized PCR-DGGE band densitometric data was performed. The PCA plot (Fig. 2b) with 52.8% variance showed that the methods based on enzymatic lysis (II, III, IV, V) and non-enzymatic lysis (I, VI) formed separate clusters (Analysis of similarity (ANOSIM), *R* = 0.814, *p* = 0.0293) while the mechanical bead beating method (VII) formed an out-group. Different diversity parameters were compared to understand the variation in eubacterial species richness and diversity (see Supplementary Table S2). Bacterial species richness (Chao1) and diversity (Shannon’s diversity) were higher in both enzyme-based methods and mechanical bead beating method for most of the food types. For subsequent analyzes, three DNA extraction methods for each food type were selected based on high DNA recovery, discrete eubacterial community profile and high eubacterial diversity. The highly diverse yeast community profiles (Fig. 2c) recovered by the selected methods underlined the importance of selecting an efficient and standard DNA extraction method for metagenomic studies. In general, the mechanical lysis by bead beating (VII) recovered higher yeast richness and diversity (see Supplementary Table S3) across all the food types.

### Eubacterial and yeast DNA recovery varied between different extraction methods

Even though a general trend of enzymatic lysis methods recovering higher DNA yield with a better depiction of microbial community was observed, superior DNA recovery does not always lead to higher species richness and diversity. So, it was imperative to check the efficacy of the extraction methods in recovering microbial DNA by quantifying its abundance in the total DNA recovered. The microbial DNA recovered by the selected three methods for each food type were quantified using qPCR assay by targeting the eubacterial SSU rRNA gene V3 region and yeast LSU rRNA gene D1/D2 region. Different extraction methods recovered different abundance of both eubacterial and yeast DNA in each food type (Table 1). One way-analysis of variance (ANOVA) revealed significant differences in the eubacterial DNA recovery (copies g^−1^ food) by different methods in fermented bamboo shoot (*p* = 0.002, F = 7.686), fermented milk (*p* = 0.01, F = 5.332) and fermented soybean (*p* = 0.02, F = 4.721). In general, the enzymatic lysis methods (II, V, VIII) recovered higher eubacterial DNA from fermented soybean, bamboo shoot and milk whereas non-enzymatic method (I) proved to be more efficient in fermented fish products (Table 1). In this study, we have introduced a parameter of “specific recovery” (SSU rRNA gene and LSU rRNA gene copies per μg of the total DNA recovered) to understand the efficiency of the extraction methods in recovering microbial DNA vis-à-vis the non-microbial background DNA. Even though the mechanical bead beating method (VII) yielded high metagenomic DNA from the fermented bamboo shoot, the recovery of eubacterial and yeast DNA were low, indicating that the method brought more background (plant) DNA than the microbial DNA (Fig. 1d, Table 1).

### MiSeq sequencing revealed the recovery of different fractions of the true eubacterial community by different methods

**Figure 3:**
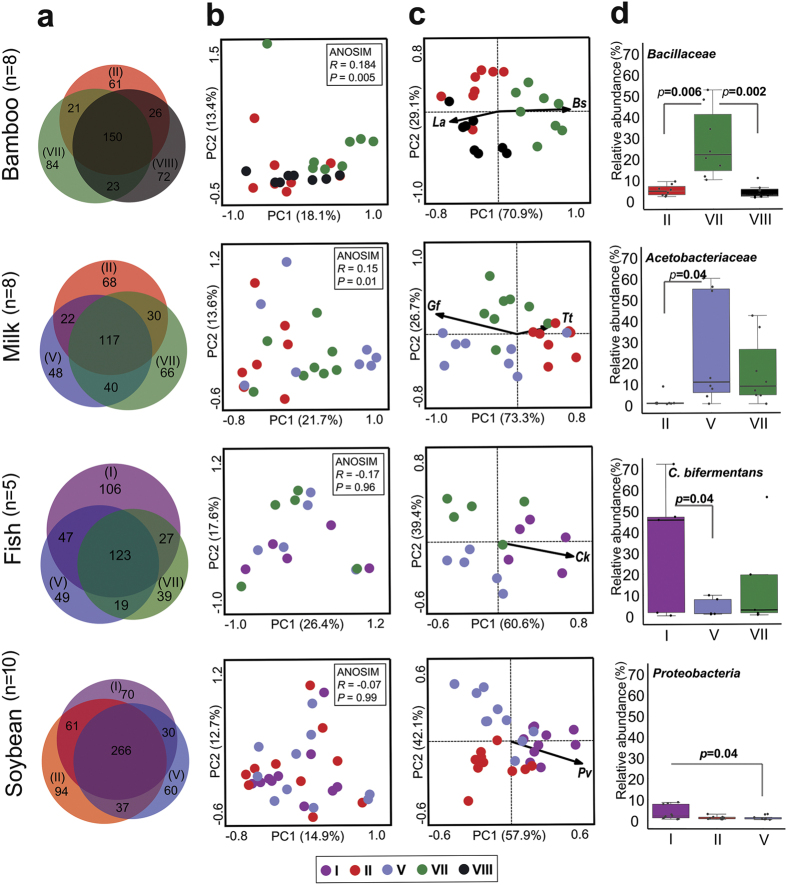
Variation in the recovery of eubacterial community structure as an effect of varied DNA extraction principles on different fermented food types. (**a**) Venn diagrams using three-fold rotationally symmetrical circles with unique and shared OTUs for different extraction methods are shown. Values in the parentheses indicate extraction methods and numbers inside each region indicate the number of unique or shared OTUs. (**b**) The canonical PCA was performed using log transformed (log x_i_ + 1) values of species-level OTU relative abundance data. A significant difference in the microbial community structure between extraction methods is highlighted by ANOSIM, performed with 10,000 replicates using Bray-Curtis distances. Circles with solid colors denote data points for each extraction method. (**c**) Species-sample biplot of RDA shows the eubacterial species direction towards different extraction methods. Solid black arrows indicate species directions towards the method and their abundance. *La*: *Lactobacillus acetotolerans*, *Bs*: *Bacillus subtilis*, *Gf*: *Gluconobacter frateurii*, *Tt*: *Thermus thermophilus*, *Ck*: *Clostridium kluyveri* and *Pv*: *Proteus vulgaris*. (**d**) The significant difference in the mean relative abundance of major taxa between extraction methods as calculated by Student’s two-tailed paired *t*-test is shown. Other significant differences at various taxonomic levels are shown in Supplementary Fig. S1, Tables S6 and S7.

**Figure 4:**
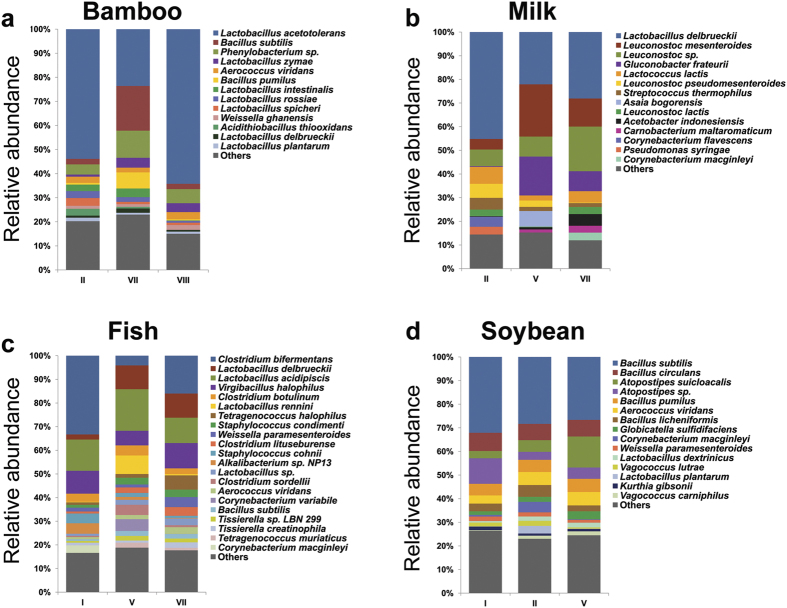
Taxon plots showing the difference in relative abundance of predominant eubacterial species recovered by different DNA extraction methods from (**a**) fermented bamboo shoot, *n* = 8; (**b**) fermented milk, *n* = 8; (**c**) fermented fish, *n* = 5 and (**d**) fermented soybean, *n* = 10. Each column represents the mean of the relative abundance of species-level OTUs analyzed by Illumina MiSeq sequencing. Taxa with less than 1% mean relative abundance across the samples studied are combined and shown as others.

Illumina MiSeq barcoded amplicon sequencing of the SSU rRNA gene V4–V5 region was performed for in-depth analysis of the observed eubacterial community differences brought out by different methods. After applying quality-filtering protocols, a total of 2,778,997 high-quality sequence reads with an average of 17,929 ± 1,139 reads per sample and an average length of 354 ± 2 bp were obtained (see Supplementary Table S4). The differences in the eubacterial community composition were compared at different taxonomic levels. The shared species-level OTUs among the three methods were low (29.9% in milk, 30% in fish, 34.3% in bamboo and 43% in soybean) (Fig. 3a). Each method recovered a substantial number of additional OTUs that were not recovered by the other methods (see Supplementary Table S5). Our results clearly indicated that employing a single DNA extraction method for studying the microbial ecology of fermented foods would result in the underestimation of at least 25% of the total OTUs. Unsupervised PCA plotting of normalized species-level OTU abundance data grouped the samples based on methods (ANOSIM, *p* < 0.01) in the fermented bamboo shoot and milk products (Fig. 3b). Redundancy analysis (RDA) biplots also indicated the significant separation of method VII in fermented bamboo shoots (Monte Carlo permutation test, *p* = 0.002, F = 2.62) and method II in fermented milk products (*p* = 0.012, F = 2.11). Species directions towards different methods are shown in Fig. 3c. Similarity percentage analysis (SIMPER) analysis showed the major species level-OTUs that contributed towards method dissimilarity (50% of the total variability) (see Supplementary Table S8). We observed significant differences in the bacterial community composition recovered by the different extraction methods (Fig. 3d, Supplementary Fig. S1). In general, lactic acid bacteria (*Lactobacillales*) were more abundant when enzymatic lysis based extraction methods were employed (see Supplementary Tables S6 and S7). However, *Bacillus (Bacillaceae*), *Gluconobacter (Acetobacteraceae*), *Clostridium (Clostridiaceae*) and *Proteobacteria* were abundantly recovered when mechanical and chemical lysis principles were applied. For example, the preferential recovery of *Bacillus subtilis* (in bamboo shoot products), *Clostridium bifermentans* (in fish products) and *Proteus vulgaris* (in soybean products) by zirconia/silica bead beating method and recovery of *Gluconobacter frateurii* by guanidium thiocyanate treatment in milk products could be mentioned. Figure 4 shows the differential recovery of bacterial community by different DNA extraction methods from the four types of naturally fermented foods.

### Recovery of higher alpha diversity after mixing of DNA extracted by different methods

**Figure 5:**
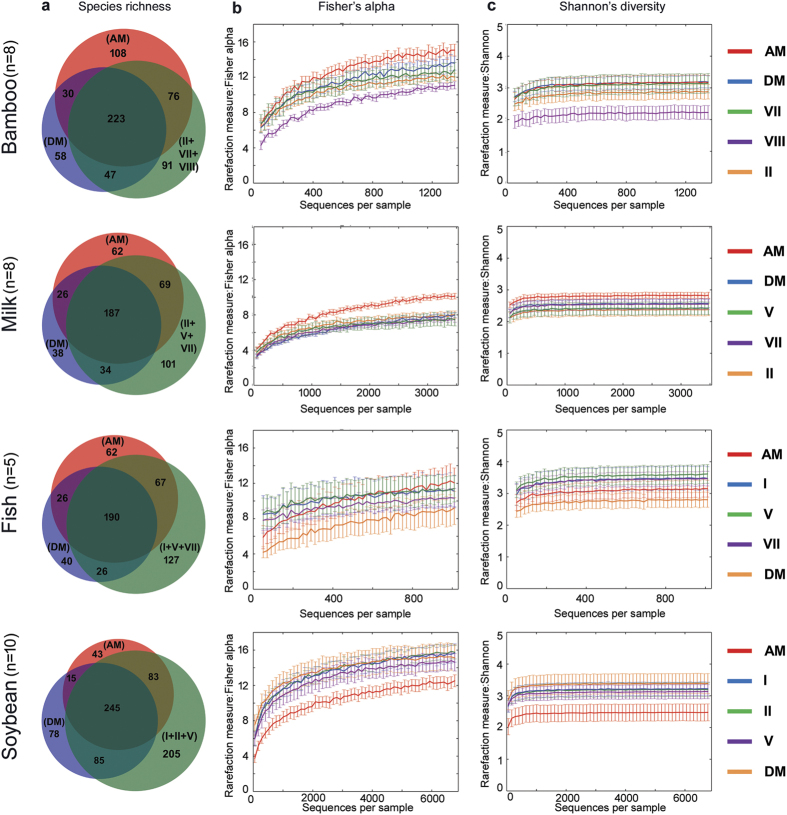
Comparison of alpha diversity differences between the two types of DNA mixing (DM, AM) and the specific individual methods in different fermented food types. **(a**) Venn diagram shows the difference in species-level OTU recovery between the mixes in comparison to the selected three individual methods for each food type. Values in the parentheses indicate extraction methods and numbers inside each region indicate the number of unique or shared OTUs. Variation among the extraction methods in the estimation of (**b**) Fisher’s alpha diversity and (**c**) Shannon’s diversity is represented as rarefaction plots. Replicates were used as mentioned elsewhere and the error bars represent standard error indicating the range of alpha diversity scores achieved at a given sampling depth.

Based on the above findings, we concluded that employing a single extraction method would lead to underestimation of the true microbial richness and diversity. A strategy was adapted to recover maximum microbial community and diversity by mixing the DNA of the individual methods in two different approaches. Equimolar mixing of metagenomic DNA (DM) and equimolar mixing of PCR amplicons (AM) generated from individual methods were subjected to MiSeq sequencing to investigate the impact of mixing on the recovery of the microbial community and diversity. Our results revealed that both types of mixing strategy (AM and DM) resulted in higher OTU recovery (53.1–68.2% in AM and 52.8–61.8% in DM of the total OTUs recovered by all the three methods together) (Fig. 5a). AM recovered higher (*p* < 0.05) species richness (Chao1) and Fisher’s alpha diversity than the other methods (Fig. 5, Supplementary Fig. S2 and Table S9) in all the food types except fermented soybean. Though Good’s coverage of 99.04 ± 0.6 in all cases indicated that our sequencing depth was sufficient to cover the high microbial diversity present in the fermented foods studied, the two mixing methods shared only 46.6–52.0% of the total OTUs recovered. Further, these mixing methods generated high number of additional OTUs (15–31.6% in AM and 9.4–20.1% in DM) which were not recovered by any of the individual methods (see Supplementary Table S10).

## Discussion

Recovering maximum metagenomic DNA from fermented food matrices is critical for meaningful and realistic analysis of their microbial ecology. Different parameters such as DNA recovery, DNA purity, microbial diversity and community structure are generally evaluated to determine the choice of DNA extraction method for different ecological niches^6^,^24^. It has been already established that methods with different DNA yield may not always bring different community structure^24^,^25^. Similarly, high DNA recovery does not always lead to high diversity^26^. In the present study, the mechanical bead beating method (VII) recovered high species diversity although it recovered low microbial DNA in both fermented bamboo shoot and milk products. Conversely, enzymatic lysis (II) recovered high microbial DNA with less diversity. This phenomenon was also observed when the raw materials of fermented bamboo shoots and fermented fish were compared with the final fermented products. The raw material exhibited high diversity with low microbial DNA yield while the fermented products recovered more microbial DNA with low diversity^6^,^27^. The low diversity might be due to the presence of excess nucleic acids from a few dominant species and hence DNA from species of the lower population was not equally amplified, resulting in lower diversity. The presence of non-microbial background DNA may also mask the amplification leading to lower diversity. In this study, we introduced an additional parameter “specific recovery” to measure the microbial DNA vis-à-vis the background food DNA while selecting the DNA extraction method. The background DNA (eukaryotic DNA from food matrix) may be removed by passing the food homogenate through column^28^ but it will lead to loss of DNA from the already lysed microbial cells. Our findings also indicated that lower microbial DNA yield recover a higher proportion of rare taxa in fermented foods. In fermented bamboo shoot and milk, mechanical method (VII) with low DNA recovery uncovered rare OTUs. Detection of rare taxa is paramount in the diagnosis of foodborne pathogens or food spoilage organisms.

Vigorous or harsh cell lysis mechanisms (bead beating, heating, sonication) are normally recommended for the ecological niche dominated by Gram-positive bacteria while gentle lysis (enzymatic lysis) is recommended for Gram-negative bacteria to achieve maximum DNA yield and diversity^9^,^29^,^30^,^31^. On the contrary, our study revealed higher recovery and diversity of lactic acid bacteria of the phylum *Firmicutes* (which are dominantly present in the naturally fermented foods) by enzymatic cell lysis compared to the harsh cell lysis principles. This finding is supported by other studies reporting the effective recovery of *Firmicutes* from saliva^32^,^33^ and faecal samples^17^ by enzymatic cell lysis. However, Hendenson *et al.*^34^ reported the abundant recovery of *Firmicutes* from rumen samples by zirconia bead beating based extraction method. This difference could be explained by the effective recovery of spore forming members of *Firmicutes viz. Bacillus*^35^,^36^ and *Clostridium*^32^,^37^ when mechanical cell lysis principles were applied. In the present study also, significantly higher recovery of *Bacillus* from bamboo shoot products and *Clostridium* from fish products was achieved when zirconia bead beating based mechanical lysis was applied. Though lactic acid bacteria are the key organisms involved in food fermentation, we emphasize the importance of using zirconia bead beating along with enzymatic lysis in fermented foods which are rich with spore forming *Bacillus* and *Clostridium.* Similar combination of lysis principles was also recommended for the recovery of *Firmicutes* from saliva^38^, faecal^39^ and vaginal samples^40^.

As evident from our results, the accuracy and reliability of in-depth sequencing studies on microbial ecology of naturally fermented foods largely depend on the DNA extraction method used. Only 30–43% of the total OTUs (extracted by three methods) were shared among the different methods. To reduce this bias, we tried successive extractions on the same cell pellet with different lysis principles but the yield and diversity were inferior to those of the enzymatic methods. Alternatively, pooling of multiple PCR amplicons derived from varied concentrations of PCR template^41^ and pooling of DNA extracted by different methods before sequencing were tried to reduce the bias^42^. Our results confirm the previous results of recovering higher number of OTUs while pooling^41^,^43^,^44^. Yet, only 50% of total OTUs were shared between the mixes (DM and AM) and generated a large number of unique and rare OTUs that were not recovered by any of the individual methods used in the mixing. However, this approach will not be suitable for quantitative studies as the relative abundance of the overlapped species will not be proportionate to the natural abundance^12^,^18^. The recovery of substantial number of additional OTUs may be partly explained by the sequencing depth and PCR biases^45^ as well as the bacterial species present in the particular ecological niche as demonstrated by using mock communities of human vagina^43^. The sequencing depth of this study (99% Good’s coverage) is sufficient to cover the microbial diversity. To overcome PCR related biases, we used a uniform concentration of template DNA at 10^7^ SSU rRNA gene copies equivalent for all the samples and chose a eubacterial-specific universal primer pair with good domain coverage (86% eubacteria coverage in ARB-Silva database, release 123) to reduce the discrimination during in-depth amplicon sequencing^46^,^47^.

Although similar comparative research on the impact of different cell lysis principles have been performed on other ecological niches, this is the first in-depth investigation on naturally fermented foods using Illumina MiSeq amplicon sequencing. Notably, we report a significantly higher recovery of lactic acid bacteria from naturally fermented foods by enzymatic cell lysis compared to other cell lysis principles. The bias generated due to the differential recovery of OTUs by different DNA extraction methods is quantitatively demonstrated here. Overcoming the biases generated by the choice of DNA extraction method, sequencing depth and PCR biases will remain a challenge for different ecological niches, even with the rapid technological advances.

## Methods

### Sampling and homogenization

Samples belonging to four types of traditional fermented foods (bamboo shoot, milk, fish and soybean) were collected from different markets of Northeast India in aseptic conditions (see Supplementary Table S11). Samples were transported in ice cool packs and stored at −80 °C within 48 h of sampling. For each food type, ten replicate samples were analyzed. Forty g of each sample was homogenized in 360 ml of sterile 0.1 M phosphate buffer saline (pH 6.4) using Stomacher 400 Circulator (Seward, UK) at 200 rpm for 2 min (soybean and fish) and 250 rpm for 3 min (bamboo). Similarly, the milk samples were homogenized in 2% sodium citrate at 200 rpm for 2 min. After homogenization, the big debris was allowed to settle down for 5 min and the homogenates were used for DNA extraction.

### Metagenomic DNA extraction

Eight different DNA extraction methods, based on one or more combination of various cell lysis principles (Table 2) were used for eubacterial and yeast metagenomic DNA extraction. Five extraction methods adapted and modified from the available literature along with two protocols developed in this study were compared with a commercial food DNA extraction kit (NucleoSpin Food, MACHEREY-NAGEL, Germany). The method VIII^6^ was used in the case of fermented bamboo shoot only. The detailed protocol of these extraction methods is included in the Supplementary Methods. The DNA extraction kit and laboratory prepared reagents were tested for the presence of contaminant DNA by DNA extraction on blank water (sterile ultrapure) before use. After confirming the negative PCR amplification (using microbial specific primers) from the above extract, the kit and reagents were used for DNA extraction from the samples. The extracted DNA was stored at −20 °C until further required.

### Quantification of total DNA and microbial DNA

The total DNA extracted from fermented bamboo shoot, milk and soybean products were quantified fluorometrically by Qubit 2.0 fluorometer (Invitrogen, Carlsbad, CA) using Qubit dsDNA BR Assay Kit (Invitrogen). Due to the low DNA yield from fish samples, high sensitive Qubit dsDNA HS Assay Kit (Invitrogen) was used. The quality of the DNA was assessed by measuring the absorbance data (A_260/280_) using spectrophotometer (NanoDrop ND-1000, USA). For eubacterial and yeast DNA quantification, 2 μl of 1:100 diluted metagenomic DNA was used for qPCR assay. The domain-specific primers, target genes and the amplification conditions are described in Supplementary Table S12. SYBR Green-based qPCR assays were performed in triplicates with no-template DNA as negative control in 20 μL assay volume containing 0.25 μM of each primer for both eubacteria and yeast and 1 × EXPRESS SYBR GreenER qPCR Supermix (Invitrogen) according to the manufacturer’s instructions. The amplifications were carried out on the Applied Biosystems 7500 standard qPCR platform. A melt curve was generated for each assay from 60 °C to 95 °C using the default conditions to check for non-specific amplification and primer-dimer formation. For each assay, a calibration curve (R^2^ > 0.99) for the calculation of eubacterial and yeast gene copies was generated on the basis of the copy number of SSU rRNA gene (2 × 10^1^–2 × 10^8^ copies) derived from the type strains *Lactobacillus plantarum* ATCC 8014 (for eubacteria) and LSU rRNA gene (2 × 10^1^–2 × 10^8^ copies) of *Candida guilliermondii* ATCC 6260 (for yeast) respectively. The calibration curve, gene copies per gram sample and gene copies per μg DNA were calculated as described previously^49^. Assay efficiencies were in the range of 0.942–0.993 for eubacteria and 0.833–0.863 for yeasts.

### Eubacteria and yeast-specific PCR-DGGE

The V3 region of the eubacterial SSU rRNA gene and D1/D2 region of the yeast LSU rRNA gene were amplified and subjected to PCR-DGGE analysis. One microlitre of the undiluted metagenomic DNA was used as the PCR template and the template-free PCR amplification was carried out for each and every set of PCR as a negative control. The PCR amplicons were subjected to 2% (w/v) agarose gel electrophoresis for checking the intactness and absence of non-specific amplification. The reproducibility of DGGE profile was tested by diluting the template DNA up to 1:1,000 (see Supplementary Note). DGGE was performed using the DCode Universal Mutation Detection System (Bio-Rad, USA) following manufacturer’s instructions. The optimum denaturing gradient range of each food type was determined by melt curve analysis. Using the optimized DGGE conditions (see Supplementary Note), the PCR amplicons were subjected to parallel DGGE for assessing the microbial community structure and diversity. The electrophoresis was performed with an initial run at 20 V for 10 min at 60 °C in all cases. After electrophoresis, the gels were stained with SYBR Gold (Invitrogen) and documented using ChemiDoc System (Bio-Rad, USA). The community profiles were analyzed using GelCompar II software v6.5 (Applied Maths, Belgium). Richness estimates and diversity indices were calculated in PAST v3.08^50^ using the DGGE band densitometric values. Based on the DNA recovery and PCR-DGGE analyzes, three extraction methods were selected and subjected to NGS analysis of the eubacterial community of each food.

### Barcoded Illumina MiSeq amplicon sequencing of eubacterial SSU rRNA gene

Barcoded Illumina MiSeq amplicon sequencing and data analysis were performed following the protocols described previously^6^ with the following modifications. The 5′ end of the reverse primer was barcoded with 12-bp error correcting Golay barcodes^51^ to enable sample multiplexing. The complete list of the forward and barcoded reverse primers used in the present study is listed in Supplementary Table S13. To enable recovery of maximum species richness and diversity, equimolar mixing of metagenomic DNA (DM) and equimolar mixing of PCR amplicons (AM) generated from individual methods were subjected to in-depth sequencing. For DM, metagenomic DNA equivalent to 10^7^ copies of SSU rRNA gene from each method were pooled and used for preparing sequence library. For AM, PCR amplicons generated from the three different methods were mixed in equimolar concentration for library preparation. MiSeq sequencing was performed at the NGS facility of Xcelris Genomics (Ahmedabad, India). The sequence data analysis using MG-RAST metagenomic analysis server^52^ and QIIME v1.8.0 bioinformatics pipeline^53^ was conducted. A total of 2,781,254 quality-filtered sequences of SSU rRNA gene V4–V5 region originating from four types of fermented foods were uploaded to MG-RAST as a part of the project ID 11495 (http://metagenomics.anl.gov/metagenomics.cgi?page=MetagenomeProject&project=11495) under the accession numbers listed in Supplementary Table S4. The quality-filtered reads were subjected to secondary quality filtering to remove non-rRNA sequences before clustering into OTUs and subsequent taxonomic assignment. Eukaryota-specific and unassigned OTUs were filtered from the OTU table before performing microbial community statistical analyzes.

### Statistical analysis

To evaluate the correlation between changes in the microbial community composition and the DNA extraction methods, multivariate PCA was performed on both the PCR-DGGE data (densitometric values of DGGE bands) and MiSeq data (relative abundance of eubacterial species-level OTUs) using Canoco software v4.52 (Wageningen University, The Netherlands). Before analysis, the data were normalized by using log transformation (log x_i_ + 1). RDA was also performed on the MiSeq data and represented as biplots. ANOSIM to test for the significant differences in the microbial community structure due to different extraction methods was performed on the MiSeq data with 10,000 permutations using Bray-Curtis distances in PAST. To identify the dominant OTUs contributing to any observed differences, SIMPER was performed using Bray-Curtis distances as implemented in PAST. Venn diagrams for graphical representation of shared and unique OTUs among the different extraction methods were created using BioVenn^54^. Any significant difference in the microbial DNA recovery or relative abundance of individual taxa between the extraction methods were tested by *p* value calculation using Student’s two-tailed paired *t*-test. *p*-value < 0.05 was considered as statistically significant. For comparison of more than two groups, one way ANOVA was performed using STATISTICA 12. The observed significant differences were represented as boxplots using BoxPlotR^55^ (http://boxplot.tyerslab.com/). For the alpha diversity analysis and generation of alpha rarefaction curves, the quality-filtered species-level OTU table was rarefied at a depth range of 50–6,850 (soybean), 50–1,355 (bamboo), 50–3,518 (curd) and 50–1,020 (fish) sequences per sample and rarefaction curves plotted using alpha_rarefactions.py script in QIIME v1.8.0. The significant difference between each method in the alpha diversity indices were calculated using the compare_alpha_diversity.py script in QIIME.

## Additional Information

**How to cite this article**: Keisam, S. *et al.* Quantifying the biases in metagenome mining for realistic assessment of microbial ecology of naturally fermented foods. *Sci. Rep.* **6**, 34155; doi: 10.1038/srep34155 (2016).

## Supplementary Material

Supplementary Information

## Figures and Tables

**Table 1:** Comparison of the efficacy of different DNA extraction methods in the recovery of eubacterial and yeast DNA from four different food types using domain-specific qPCR assays.

Food type	Method	Bacterial DNA recovery		Yeast DNA recovery	
		Total recovery	Specific recovery	Total recovery	Specific recovery
Soybean	I	9.94 ± 0.15	9.15 ± 0.05	6.68 ± 1.52	6.21 ± 1.67
	II	10.61 ± 0.17 (I)	9.27 ± 0.15	6.75 ± 0.62	5.15 ± 0.64
	V	10.79 ± 0.10 (I)	9.20 ± 0.04	7.42 ± 0.54	5.79 ± 0.59
Milk	II	10.43 ± 0.10 (VII)	9.45 ± 0.04 (V)	10.22 ± 0.31	9.23 ± 0.35
	V	10.50 ± 0.16	9.29 ± 0.03	10.33 ± 0.27	9.12 ± 0.23
	VII	10.07 ± 0.09	9.22 ± 0.08	9.63 ± 0.21	9.01 ± 0.35
Fish	I	9.45 ± 0.30 (VII)	9.10 ± 0.28	7.05 ± 0.30	6.80 ± 0.27
	V	8.88 ± 0.47	8.99 ± 0.27	6.75 ± 0.25	6.74 ± 0.18
	VII	8.95 ± 0.42	8.96 ± 0.28	7.35 ± 0.50	7.59 ± 0.38
Bamboo	II	9.04 ± 0.12 (VII)	8.36 ± 0.16 (VII)	7.84 ± 0.14 (VII, VIII)	7.16 ± 0.12 (VII)
	VII	7.54 ± 0.23	6.80 ± 0.22	6.79 ± 0.25	6.04 ± 0.23
	VIII	9.13 ± 0.13 (VII)	8.44 ± 0.17 (VII)	7.40 ± 0.15 (VII)	6.88 ± 0.24 (VII)

Total recovery is indicated as log rRNA gene copies g^−1^ of food and specific recovery is indicated as log rRNA gene copies μg^−1^ of DNA. Data represents the arithmetic mean ± standard error of the mean of 10 independent replicates. Methods with significantly (*p* < 0.05, Student’s two-tailed paired *t*-test) lower microbial DNA recovery as compared to a particular method are indicated in the parentheses.

**Table 2:** A summary of the different metagenomic DNA extraction methods used in this study.

Method code	Cell lysis principles	Lysis agents with final concentration	Reference
I	Mechanical	Zirconia/silica beads	1
	Chemical	0.8% SDS and 1.2% Triton X-100	
II	Enzymatic	(333 KU lysozyme, 166 U mutanolysin and 133 U lyticase) per g sample	48
	Chemical	0.5% SDS	
III	Enzymatic	(333 KU lysozyme, 166 U mutanolysin and 133 U lyticase) per g sample	This study
	Chemical	0.6% SDS	
	Mechanical	Zirconia/silica beads	
	Heating	95 °C	
IV	Enzymatic	(333 KU lysozyme, 166 U mutanolysin and 133 U lyticase) per g sample	This study
	Sonication	50/60 Hz, 2.0 amplitude for 2 cycles (30 s pulse on, 5 s pulse off)	
	Heating	95 °C	
V	Enzymatic	333 KU lysozyme, 166 U mutanolysin and 133 U lyticase per g sample	16
	Chemical	5 mM guanidium thiocyanate and 0.05% sarkosyl	
VI	Chemical	NucleoSpin Food kit, MACHEREY-NAGEL	—
VII	Chemical	0.4% SDS	17
	Mechanical	Zirconia/silica beads	
	Heating	95 °C	
VIII	Enzymatic	(5 KU lysozyme, 25 U mutanolysisn and 20 U lyticase) per g sample 0.2% SDS and 0.4% Triton X-100	6

See Supplementary Methods for detailed protocol.
