# Supplementary material for: Quantifying the biases in metagenome mining for realistic assessment of microbial ecology of naturally fermented foods
Source: Sci Rep. 2016 Sep 27;6:34155. doi: 10.1038/srep34155 (PMC5037447; doi:10.1038/srep34155)
Supplement: Supplementary Information [file srep34155-s1.pdf]

Supplementary Information

Title: Quantifying the biases in metagenome mining for realistic assessment of microbial ecology of naturally fermented foods.

Authors: Santosh Keisam, Wahengbam Romi, Giasuddin Ahmed and Kumaraswamy Jeyaram*

Contents Summary

Supplementary Note

Optimization of PCR-DGGE conditions for studying the eubacterial and yeast community structure of four different food types are described here.

SupplementaryFigures:

Fig. S1. Significant difference in the relative abundance of specific eubacterial OTUs at phylum and family level recovered by different DNA extraction methods in fermented (**a**) milk and **(b)** bamboo shoot products.

Fig. S2. Variation among the extraction methods in the estimation of **(a)** Chao species richness, **(b)** Shannon's equitability and **(c)** Shannon's diversity is represented as rarefaction plots. Replicates were used as mentioned elsewhere and the error bars represent standard error indicating the range of alpha diversity scores achieved at a given sampling depth.

Supplementary Tables:

Table S1. Student's paired two-tailed *t*- test (*P*-values) for comparison of mean DNA yield of different DNA extraction methods for four different food types.

Table S2. Comparison of PCR-DGGE based eubacterial alpha diversity estimates of four fermented food types among different extraction methods.

Table S3. Comparison of PCR-DGGE based yeast alpha diversity estimates of four fermented food types among different extraction methods.

Table S4. Summary statistics of Illumina MiSeq sequencing data as analysed in MG-RAST web-server.

Table S5. Additional species-level OTUs extracted by each specific method from four food types.

Table S6. Mean relative abundance of genus-level eubacterial OTUs recovered by different extraction methods (I, II, V, VII, and VIII) and significant differences of the means in three food types.

Table S7. Mean relative abundance of species-level eubacterial OTUs recovered by different extraction methods (I, II, V, VII and VIII) and significant differences of the means in four food types

Table S8. Summary of SIMPER analysis showing species-level OTUs that contribute to 50% variability in the microbial community structure for each food type recovered by different methods (I, II, V, VII and VIII).

Table S9. Sequence diversity and library coverage estimations of Illumina MiSeq sequencing.

Table S10. Additional species-level OTU extracted by the combination of three methods and the two types of mixture from four food types. For the comparsion of AM and DM with the individual extraction methods, the data obtained from each individual method were combined into a single OTU table.

Table S11. Details of naturally fermented foods collected from Northeast India for the present study.

Table S12. List of primers and PCR conditions used for PCR-DGGE and qPCR analysis.

Table S13. List of forward and barcoded reverse primers used for amplification of eubacterial SSU rRNA gene V4-V5 region for multiplexed Illumina MiSeq sequencing.

Supplementary Methods

The detailed protocol of the different metagenomic DNA extraction methods used in this study are recorded here.

Supplementary Note

An accurate analysis of PCR-DGGE profile depends on obtaining high-resolution band separation with minimum co-migration of bands. So, PCR-DGGE conditions (denaturing gradient and volt-hr) for each food type was optimized as different food types have different microbial community structure. To optimise the denaturing gradient a melt curve analysis was performed using perpendicular DGGE, using 8% (w/v) polyacrylamide gels (acrylamide/bisacrylamide, 37.5:1) with a denaturing gradient range of 0%–80% (100% denaturant corresponds to a mixture of 1 M urea and 40% (v/v) formamide). The electrophoresis was carried out at 100 V for 1 h at 60 °C. Narrow denaturing gradient range (27-52%) in the case of fermented milk and wider range (30-60%) in the case of fermented fish (Figure A) during perpendicular DGGE analysis indicated the importance of optimizing DGGE conditions.

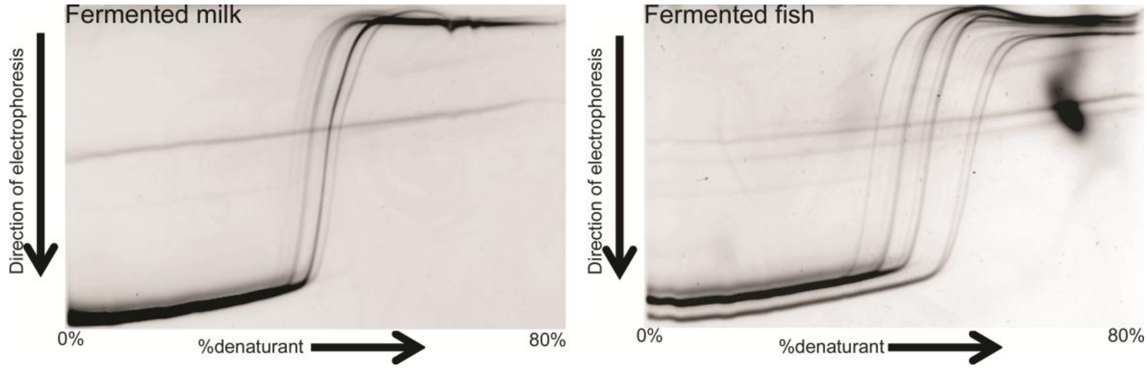

Figure A. Optimization of denaturing gradient for PCR-DGGE analysis of fermented milk and fish products using perpendicular DGGE at 100 V for 1 h at 60 ˚C. Solid arrows indicate the direction of electrophoresis and denaturing gradient.

To investigate the potential bias associated with using different amount of template DNA in the PCR-DGGE analysis, metagenomic DNA from fermented soybean and milk samples were used at different dilutions (2x, 10x and 100x diltutions) for comparative profile

analysis with the undiluted profile. Since there were no significant differences in the microbial community profile (Figure B) when observed at different dilutions, undiluted DNA was used for subsequent analyzes. Using the optimized conditions (Table A), PCR-DGGE was performed using universal eubacterial and yeast primers with high domain coverage and specificity (eubacteria-81%; yeast-66% based on ARB-SILVA, release 123).

Contrary to earlier finding[1], we found that the dilution of PCR template during PCR-DGGE analysis did not lead to a contrasting view of the microbial community structure. As long as the PCR is not inhibited, which may happen due to high template concentration, the resulting products may not show any significant difference in their community profile. The dye used for gel staining was also optimized as DGGE analysis is based on the visual detection of bands. Out of the three dyes used (ethidium bromide, silver staining, SYBR), SYBR Gold was found to be the most effective as reported in other study[2] (Figure C). The amplicons used in both eubacterial and yeast PCR-DGGE were generated using an extended elongation time as this prevents overestimation of the microbial diversity by reducing the formation of artifactual double bands[3]. During the eubacterial DGGE optimization, we observed that fermented milk products had a narrow denaturing gradient range while the fermented fish products had a wider gradient range. This can be explained by the fact that fermented milk products are dominated by closely related species of lactic acid bacteria while fermented fish products are dominated by species belonging to phylogenetically distinct group of bacteria.

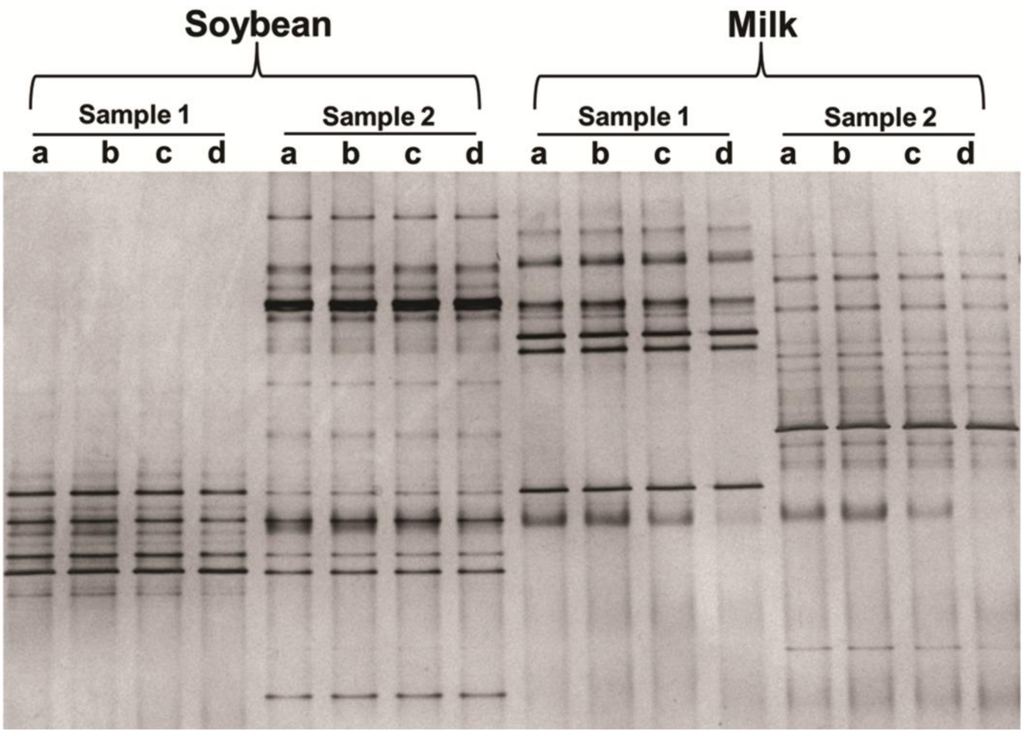

Figure B. Reproducibility in the eubacterial community PCR-DGGE fingerprints of fermented soybean and milk at different dilutions of the metagenomic DNA. Lanes: **a**-undiluted; **b**-2x dilution; **c**-10x dilution and **d**-100x dilution.

Table A. Optimized DGGE conditions for studying eubacterial and yeast community structure of four different food types.

Food type	*Denaturing gradient (%)*	*Electrophoresis conditions*	
		Voltage	*Runtime*
Bacteria			
Fermented bamboo shoot	30–60	150 V	5 h
Fermented milk	27–52	140 V	4 h
Fermented fish	30–60	160 V	5 h
Fermented soybean	25–55	140 V	5 h
Yeast			
All food types	31–44	150 V	3 h 45 min

100% denaturant corresponds to 7M urea and 40% (wt/vol) formamide.

Electrophoresis was performed in 0.75 mm x 16 cm x 16 cm, 8% (wt/vol) polyacrylamide gels.

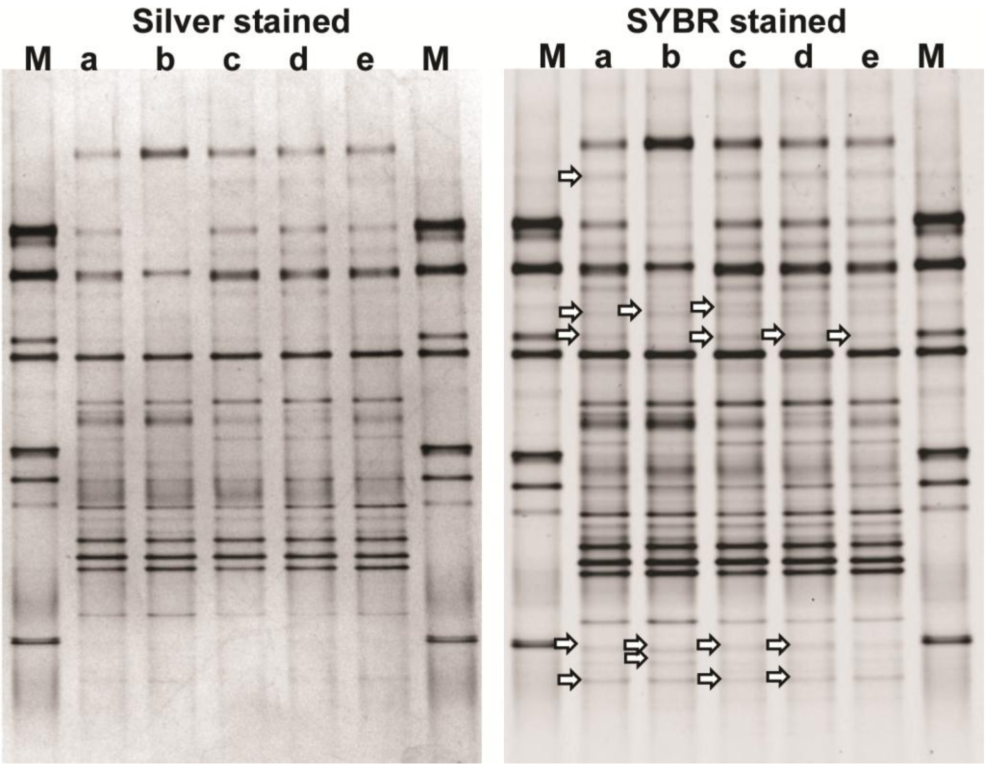

Figure C. Comparison of silver-stained gels and SYBR Gold-stained gels for PCR-DGGE analysis. Bands which are detected only in SYBR-stained gels are indicated with arrows. Lanes: M- DNA ladder, a–e- Metagenomic DNA samples.

References

1. Garcia-Armisen, T. *et al.* Diversity of the total bacterial community associated with Ghanaian and Brazilian cocoa bean fermentation samples as revealed by a 16S rRNA gene clone library. *Appl. Microbiol. Biotechnol.* **87,** 2281-2292 (2010).
2. Vanhoutte, T., Huys, G., Brandt, E. & Swings, J. Temporal stability analysis of the microbiota in human feces by denaturing gradient gel electrophoresis using universal and group-specific 16S rRNA gene primers. *FEMS Microbiol. Ecol*. **48,** 437-446, (2004).
3. Janse, I., Bok, J. & Zwart, G. A simple remedy against artifactual double bands in denaturing gradient gel electrophoresis. *J. Microbiol. Methods* **57,** 279-281 (2004).

Supplementary Methods

The detailed protocol of the different metagenomic DNA extraction methods used in this study are recorded here.

Method I

- Transfer 1.5 ml of the homogenate to a sterile 2 ml screw-cap tube containing 0.5 g of zirconia/silica beads (0.1 mm) and 4 glass beads (2 mm).
- Centrifuge the tube at 4 °C for 10 min at 14 000 x g and discard the supernatant. Add 1 ml of petroleum ether: hexane (1:1) to the pellet and incubate at room temperature for 10 min.
- Centrifuge it for 10 min at 14 000 x g and resuspend the pellet in 150 µl of proteinase K buffer [50 mM Tris-Cl, 10 mM EDTA (pH 8), 0.5% (w/v) SDS]. Add 25 µl of proteinase K (25 mg/ml) to the mixture and incubate overnight at 65 °C.
- Add 150 µl of 2X breaking buffer [4% Triton X-100 (v/v), 2% (w/v) SDS, 200 mM NaCl, 20 mM Tris (pH 8), 2 mM EDTA (pH 8)].
- Add 300 µl of phenol (pH 8) to the above mixture and vortex three times at maximum speed with an interval of 10 s for 1 min in a bead beater. Centrifuge at 4 °C for 10 min at 12 000 x g and collect the aqueous phase.
- Purify it twice with chloroform:isoamyl alcohol mixture (24:1), each time with centrifugation at 4 °C for 10 min at 14 000 x g.
- Add 1 ml of absolute ethanol to the aqueous layer, mix gently and centrifuge at 4 °C for 15 min at 14 000 x g.
- Wash the pellet twice with 70% ethanol and precipitate the DNA by centrifuging at 4 °C for 15 min at 14 000 x g after each wash. Dry the pellet at room temperature and dissolve it in 50 µl of TE buffer (10 mM Tris, 1 mM EDTA).

Method II

- Transfer 1.5 ml of the homogenate to a sterile 2 ml centrifuge tube and centrifuge for 10 min at 18 000 x g.
- To the pellet, add 100 µl of lysozyme (50 KU/100 µl) in TES buffer [50 mM Tris, 1 mM EDTA (pH 8), 8.7% sucrose], 5 µl of mutanolysin (5 U/µl) and 2 µl of lyticase (10 U/µl). Vortex it for 1 min and incubate at 37 °C for 1 h.
- Add 20 µl of proteinase K (25 mg/ml) and incubate again at 65 °C for 1 h. After incubation, add 300 µl of prewarmed (65 °C) NTS buffer (0.2 M NaCl, 0.1 M Tris, 2% SDS) and further incubate at 65 °C for 10 min.
- Purify the sample once with phenol (pH 8) and twice with chloroform:isoamyl alcohol mixture (24:1), each time with centrifugation at 4 °C for 15 min at 15 000 x g.
- Add 0.7 volume of isopropanol to the aqueous layer, mix gently and centrifuge at 4 °C for 15 min at 15 000 x g.
- Wash the pellet twice with 70% ethanol and precipitate the DNA by centrifuging at 4 °C for 15 min at 15 000 x g after each wash. Dry the pellet at room temperature and dissolve it in 50 µl of TE buffer (10 mM Tris, 1 mM EDTA).

Method III

- Transfer 1.5 ml of the homogenate to a sterile 2 ml screw-cap tube containing 0.5 g of zirconia/silica beads (0.1 mm) and 4 glass beads (2 mm). Centrifuge for 10 min at 18 000 x g.
- To the pellet, add 100 µl of lysozyme (50 KU/100 µl) in TES buffer [50 mM Tris, 1 mM EDTA (pH 8), 8.7% sucrose], 5 µl of mutanolysin (5 U/µl) and 2 µl of lyticase (10 U/µl). Vortex it for 1 min and incubate at 37 °C for 1 h.
- Add 20 µl of Proteinase K (25 mg/ml) and incubate again at 65 °C for 1 h. After incubation, add 300 µl of prewarmed NTS buffer [0.4 M NaCl, 0.2 M Tris and 2.75% (w/v) SDS] and further incubate at 65 °C for 10 min.

- Treat sample in fast prep at room temperature for 3 x 1 min (cool samples on ice in between for 30 s). Heat the sample at 95 °C for 15 min. Mix the samples by shaking every 5 min.
- Purify once with phenol (pH 8) and twice with chloroform:isoamyl alcohol mixture (24:1) each time with centrifugation at 4 °C for 15 min at 15 000 x g.
- Add 0.7 volume of isopropanol to the aqueous layer, mix gently and centrifuge at 4 °C for 15 min at 15 000 x g.
- Wash the pellet twice with 70% ethanol and precipitate the DNA by centrifuging at 4 °C for 15 min at 15 000 x g after each wash. Dry the pellet at room temperature and dissolve it in 50 µl of TE buffer (10 mM Tris, 1 mM EDTA).

Method IV

- Transfer 1.5 ml of the homogenate to a sterile 2 ml centrifuge tube and centrifuge for 10 min at 18 000 x g.
- To the pellet, add 100 µl of lysozyme (50 KU/100µl) in TES buffer [50 mM Tris, 1 mM EDTA (pH 8), 8.7% sucrose], 5 µl of mutanolysin (5 U/µl) and 2 µl of lyticase (10 U/µl). Vortex for 1 min and incubate at 37 °C for 1 h.
- Add 20 µl of Proteinase K (25 mg/ml) and incubate at 65 °C for 1 h. After incubation, add 300 µl of TES buffer (50 mM Tris, 1 mM EDTA, 8.7% sucrose) and mix gently.
- Treat with sonication using Misonix 3000 sonicator at amplitude of 2.0 for 2 cycles (30 s pulse on, 5 s pulse off). Keep the samples on ice during sonication.
- After sonication, heat the samples at 95 °C for 15 min. Mix the samples by shaking every 5 min.
- Purify the sample once with phenol (pH 8) and twice with chloroform:isoamyl alcohol mixture (24:1), each time with centrifugation at 4 °C for 15 min at 15 000 x g.
- Add 0.7 volume of isopropanol to the aqueous layer. Mix gently and store at -20 °C for 1h. Centrifuge at 4 °C for 15 min at 15 000 x g.

- Wash the pellet twice with 70% ethanol and precipitate the DNA by centrifuging at 4 °C for 15 min at 15 000 x g after each wash. Dry the pellet at room temperature and dissolve it in 50 µl of TE buffer (10 mM Tris, 1 mM EDTA).

<u>Method V</u>

- Transfer 1.5 ml of the homogenate to a sterile 2 ml centrifuge tube and centrifuge for 10 min at 18 000 x g.
- To the pellet, add 400 µl of TES buffer (50 mM Tris, 1 mM EDTA, 8.7% sucrose), 100 µl of lysozyme (50 KU/100 µl TES), 5 µl of mutanolysin (5 U/µl) and 20 U of lyticase (2 µl of 10 U/µl). Vortex it for 1 min and incubate at 37 °C for 1 h.
- Add 20 µl of Proteinase K (25 mg/ml) and incubate at 65 °C for 1 h.
- Add 500 µl of GES reagent (5 M guanidine thiocyanate, 100 mM EDTA, and 0.5% sarkosyl) to each tube and cool on ice for 5 min.
- Add 250 µl of cold 7.5 M ammonium acetate. Mix the two phases by shaking gently and cool on ice for 10 min.
- Add 600 µl of chloroform:isoamyl alcohol (24:1) to the tube. Mix properly and centrifuge at 15 000 x g for 10 min at 4 °C.
- Add 0.7 volume of isopropanol to the aqueous layer. Mix gently and store at -20 °C for 1 h. Centrifuge at 4 °C for 15 min at 15 000 x g.
- Pellet wash with 70% ethanol twice and DNA precipitated by centrifuging at 4 °C for 15 min at 15 000 x g.
- Dry the pellet at room temperature and dissolve it in 50 µl of TE buffer (10mM tris, 1mM EDTA)

Method VI

As per manufacturer’s instructions for NucleoSpin Food (MACHEREY- NAGEL, Germany. Cat no. 740945)

Method VII

Kit requirement: QIAamp DNA Stool Mini Kit (Qiagen, Cat no. 51504)

- Transfer 1.5 ml of the homogenate to a sterile 2 ml screw-cap tube containing 0.5 g of zirconia/silica beads (0.1 mm) and 4 glass beads (2 mm). Centrifuge for 10 min at 18 000 x g.
- Discard the supernatant and add 1 ml of lysis buffer (500 mM NaCl, 50 mM Tris-HCl (pH 8), 50 mM EDTA, 4% SDS). If buffer is precipitated heat at 70 °C.
- Treat the samples in FastPrep at room temperature for 3 x 1 min (cool samples on ice in between). Heat at 95 °C for 15 min and mix the samples by shaking every 5 min.
- Centrifuge at 4 °C for 10 min at 15 000 x g and transfer the supernatant to a fresh 2 ml centrifuge tube. Add 300 µl of fresh lysis buffer to the tube and repeat steps 3-4, then pool the supernatants.
- Add 260 µl of 10 M ammonium acetate to each tube, mix well, and incubate on ice for 5 min. Centrifuge at 4 °C for 10 min at 15 000 x g.
- Transfer the supernatant to 1.5 ml tube and add 0.7 volume of isopropanol to the aqueous layer. Mix gently, incubate on ice for 30 min and centrifuge at 4 °C for 15 min at 15 000 x g.
- Wash the pellet with 70% ethanol and precipitate the DNA by centrifuging at 4 °C for 15 min at 15 000 x g. Dry the pellet at room temperature and dissolve it in 100 µl each of TE buffer (10 mM Tris, 1 mM EDTA). Leave at 4 °C overnight and pool the two aliquots.
- Add 2 µl of DNase-free RNase (10 mg/ml) and incubate at 37 °C for 15 min.
- Add 15 µl of proteinase K and 200 µl of Buffer AL mix well and incubate at 70 °C for 10 min.
- Add 200 µl of absolute ethanol and mix well. Transfer to a QIAmp column and centrifuge for 1 min at 13 000 x g.
- Discard the flow through, add 500 µl of Buffer AW1 and centrifuge at room temperature for 1 min at 13 000 x g.

- Discard the flow through, add 500 µl of Buffer AW2 and centrifuge at room temperature for 1 min at 13 000 x g.
- Dry the column by centrifugation at room temperature for 1 min. Add 100 µl of Buffer AE and incubate at room temperature for 1 min. Then centrifuge at 13 000 x g for 1 min.
- Add fresh 100 µl of buffer AE, incubate for 1 min at room temperature and centrifuge at 13 000 x g for 1 min. Pool the two aliquots.

Method VIII

- Transfer 10 ml of the homogenate to a 50 ml centrifuge tube. Centrifuge at 2 500 x g for 10 min at 4 °C.
- Wash the cell pellet with sterile distilled water and transferred to a 1.5 ml centrifuge tube. Centrifuge at 15 000 x g for 10 min at 4 °C.
- Resuspend the cell pellet in 150 µl of TES buffer (50 mM Tris, 1 mM EDTA, 8.7% sucrose, pH 8). Add 10 µl of 50 KU/100 µl lysozyme, 5 µl of 5 U/µl mutanolysin, 2 µl of 10 U/ l and 90 µl of sterile distilled water. Incubate at 37 °C for 1 h.
- Add 300 µl of hot (65 °C) 2X breaking Buffer (10 mM Tris.Cl, pH 8; 100 mM NaCl; 1 mM EDTA, pH 8.0; 1% w/v SDS; 2% v/v Triton X-100) and 10 µl of 25 mg/ml proteinase K. Mix by vortexing and incubate overnight at 65 °C.
- Add 100 µl of 5 M NaCl and mix gently and thoroughly by tapping the ends of the tubes. Add 80 µl of CTAB/NaCl solution (10% CTAB/0.7 M NaCl) and mix by vortexing. Incubate at 65 °C for 10 min.
- Add equal volume of phenol:chloroform:isoamyl alcohol mixture (25:24:1) (pH 8). Mix vigorously by vortexing at maximum speed for 1 min and centrifuge at 15 000 x g for 10 min at 4 °C.
- Collect the aqueous layer and similarly extract twice with chloroform:isoamyl alcohol mixture (24:1).

- Add 0.1 volume of 3 M Na-acetate (pH 5.2) to the aqueous layer and mix gently and thoroughly by inverting the tubes. Add 1 volume of isopropanol and keep at -20 °C for 1h.
- Centrifuge at 16 000 x g for 20 min at 4°C. Discard the supernatant and wash the pellet twice with 70% ethanol.
- Dry the pellet at room temperature and dissolve it in 50 µl of TE buffer (10 mM Tris, 1 mM EDTA).

Supplementary Figures

Fig. S1

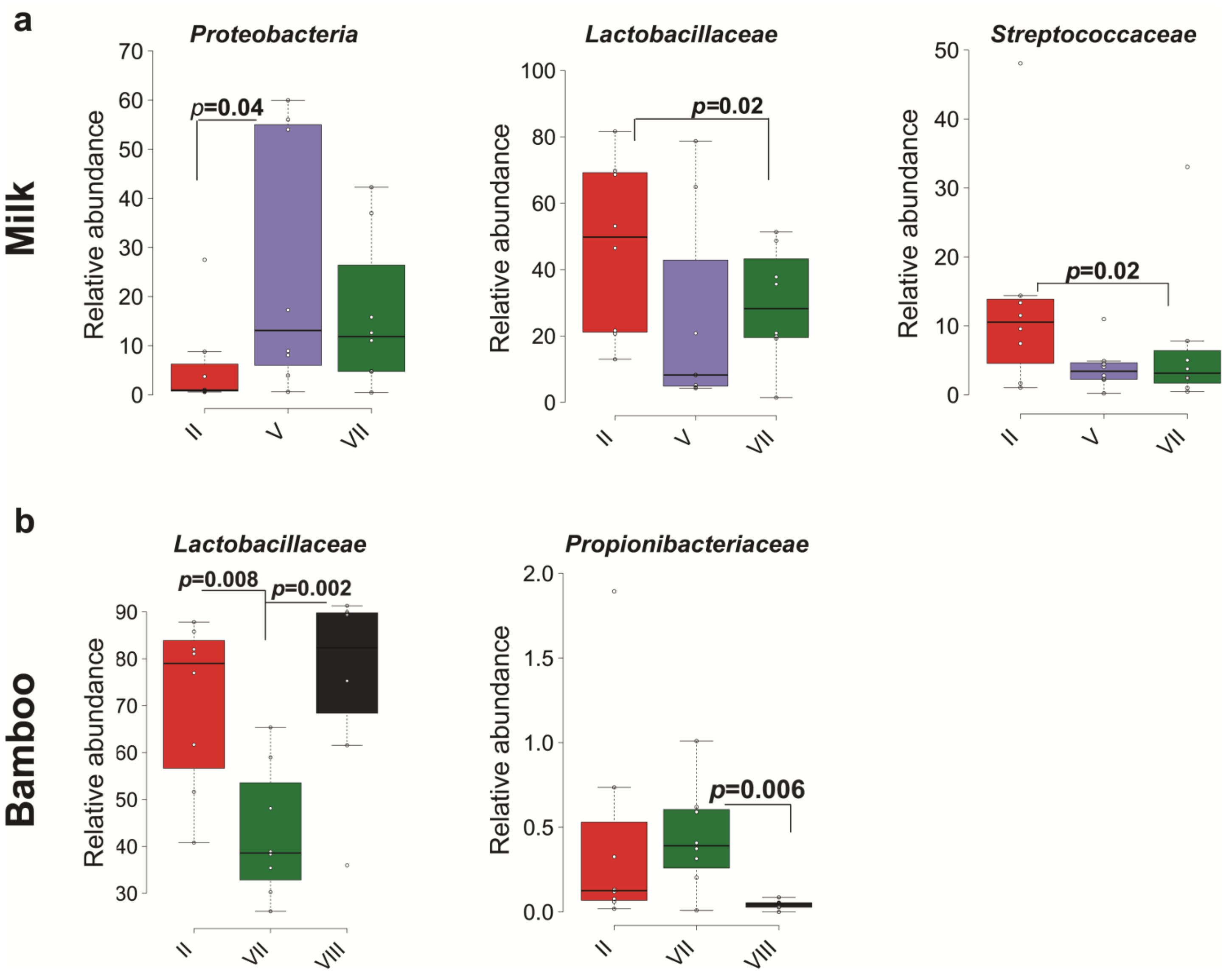

Fig.S1. Significant difference in the relative abundance of specific eubacterial OTUs at phylum and family level recovered by different DNA extraction methods in fermented (a) milk and (b) bamboo shoot products (n=8 each).

Fig. S2

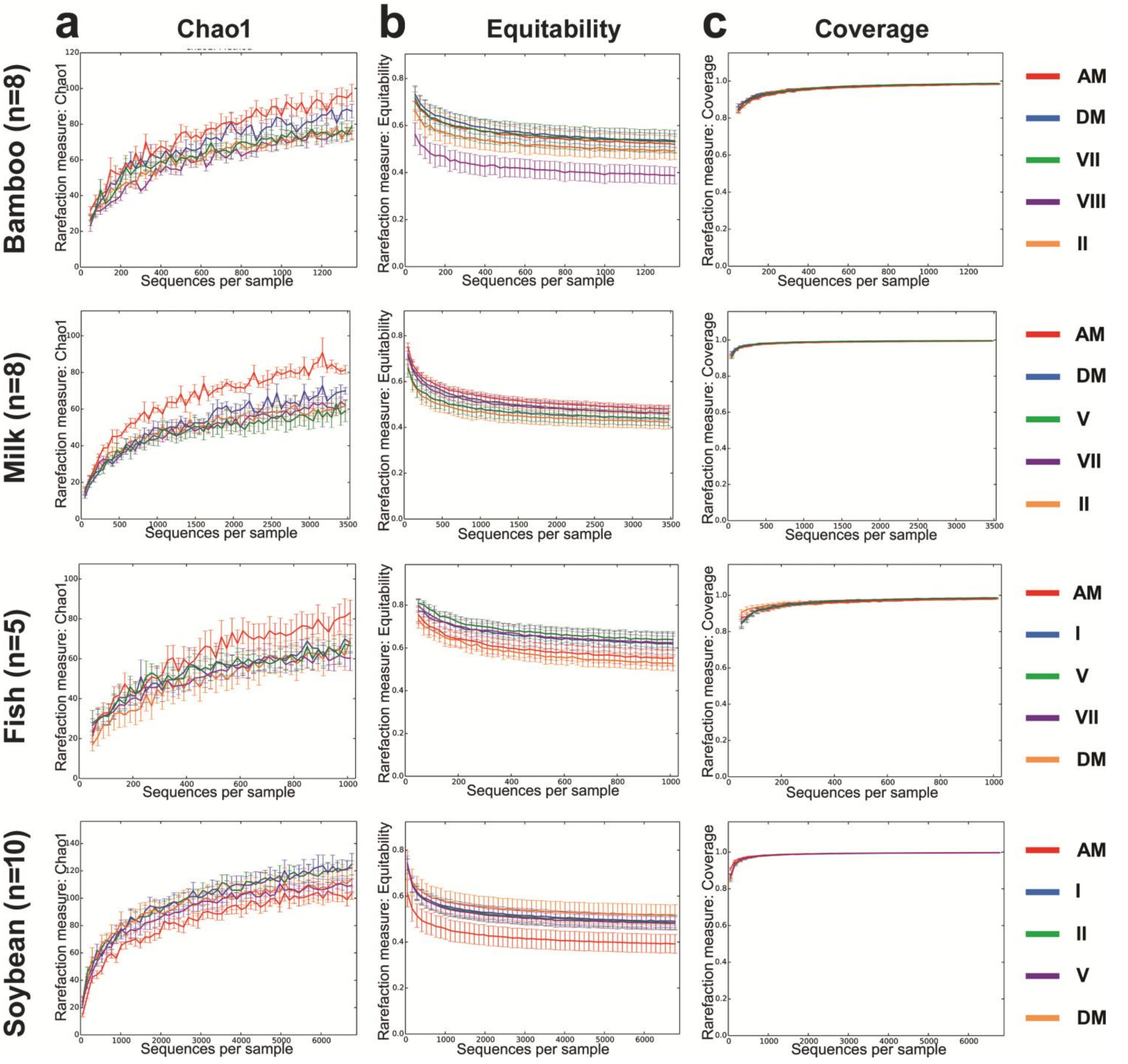

Fig. S2. Variation among the extraction methods in the estimation of (a) Chao species richness, (b) Shannon's equitability and (c) Shannon's diversity is represented as rarefaction plots. Replicates were used as mentioned elsewhere and the error bars represent standard error indicating the range of alpha diversity scores achieved at a given sampling depth.

Supplementary Tables

Table S1. Student's paired two-tailed *t*- test (*P*-values) for comparison of mean DNA yield of different DNA extraction methods for four different food types. DNA yield was estimated by fluorometric quantification using Qubit fluorometer. $P< 0.05$ is indicated in bold.

Fermented soybean						
Method	II	III	IV	V	VI	VII
I	**0.018**	0.413	0.126	**0.001**	0.300	0.328
II		**0.021**	**0.014**	0.212	**0.014**	**0.023**
III			0.306	**0.001**	0.715	0.060
IV				**0.001**	0.574	**0.027**
V					**0.001**	**0.001**
VI						**0.005**
Fermented milk						
Method	II	III	IV	V	VI	VII
I	0.123	**0.021**	**0.008**	0.278	**0.002**	**0.011**
II		0.066	**0.029**	0.090	**0.031**	**0.035**
III			0.999	**0.005**	0.993	0.506
IV				**0.003**	0.993	0.674
V					**0.017**	**0.014**
VI						0.599
Fermented fish						
Method	II	III	IV	V	VI	VII
I	**0.027**	**0.030**	**0.035**	0.053	**0.033**	**0.035**
II		0.087	0.233	0.786	0.086	0.331

III			0.843	**0.018**	0.229	0.058
IV				0.082	0.442	0.464
V					**0.009**	0.408
VI						0.073

Fermented bamboo shoot

Method	II	III	IV	V	VI	VII	VIII
I	0.112	**0.035**	**0.004**	0.409	0.138	0.088	0.918
II		**0.007**	**0.005**	0.655	0.767	0.959	0.181
III			0.107	0.214	0.178	0.251	**0.031**
IV				0.103	**9.0E-06**	**7.2E-07**	**0.038**
V					0.885	0.734	0.422
VI						0.567	0.336
VII							0.336

Table S2. Comparison of PCR-DGGE based eubacterial alpha diversity estimates of four fermented food types among different extraction methods. Alpha diversity indices were calculated in PAST with 10,000 bootstrap replications. Buzas and Gibson's evenness is calculated by e^H/S, where H is Shannon diversity and S is number of taxa.

**Food type**	**Method**	**Chao1**	**Fisher alpha**	**Dominance**	**Shannon diversity**	**Evenness**
Fermented bamboo shoot	I	15.00	2.57	0.12	2.37	0.72
	II	18.90	3.22	0.10	2.56	0.70
	III	17.20	2.96	0.11	2.47	0.70
	IV	18.90	3.29	0.11	2.54	0.70
	V	17.40	2.98	0.10	2.50	0.71
	VI	16.00	2.72	0.12	2.43	0.72
	VII	14.80	2.67	0.18	2.14	0.62
	VIII	16.30	2.78	0.13	2.40	0.70
Fermented milk	I	17.60	2.96	0.10	2.55	0.74
	II	18.30	3.11	0.09	2.59	0.75
	III	18.30	3.06	0.09	2.64	0.77
	IV	17.60	3.02	0.10	2.54	0.75
	V	18.00	3.03	0.09	2.63	0.78
	VI	17.10	2.87	0.10	2.54	0.75
	VII	18.90	3.22	0.09	2.64	0.76

Fermented fish	I	16.50	2.83	0.13	2.39	0.69
	II	17.70	3.05	0.11	2.52	0.73
	III	15.80	2.86	0.12	2.40	0.71
	IV	15.70	2.62	0.13	2.34	0.68
	V	17.60	3.01	0.11	2.52	0.73
	VI	16.60	2.89	0.14	2.36	0.67
	VII	17.40	3.02	0.12	2.43	0.71
Fermented soybean	I	21.80	3.74	0.09	2.65	0.67
	II	22.70	3.84	0.09	2.71	0.68
	III	22.60	3.76	0.09	2.72	0.71
	IV	21.60	3.64	0.10	2.63	0.67
	V	22.50	3.73	0.09	2.70	0.69
	VI	18.70	3.17	0.10	2.56	0.71
	VII	21.80	3.66	0.08	2.72	0.72

Table S3. Comparison of PCR-DGGE based yeast alpha diversity estimates of four fermented food types among different extraction methods. Alpha diversity indices were calculated in PAST with 10,000 bootstrap replications. Buzas and Gibson's evenness is calculated by e^H/S, where H is Shannon diversity and S is number of taxa.

**Food type**	**Method**	**Chao1**	**Fisher alpha**	**Dominance**	**Shannon diversity**	**Evenness**
Fermented bamboo shoot	II	9.50	1.61	0.20	1.85	0.69
	VII	12.10	2.17	0.20	1.96	0.65
	VIII	9.50	1.62	0.22	1.81	0.69
Fermented milk	II	10.80	1.90	0.19	1.93	0.73
	V	10.80	1.82	0.17	2.01	0.74
	VII	12.00	2.04	0.15	2.12	0.73
Fermented fish	I	7.78	1.43	0.32	1.57	0.70
	V	8.22	1.49	0.27	1.58	0.64
	VII	8.67	1.55	0.29	1.63	0.66
Fermented soybean	I	12.20	2.41	0.21	1.91	0.61
	II	6.90	1.25	0.36	1.39	0.65
	V	7.40	1.43	0.41	1.32	0.56

Table S4. Summary statistics of Illumina MiSeq sequencing data as analysed in MG-RAST web-server. During primary QC, joined and demultiplexed sequences were quality-filtered based on length, number of ambiguous bases and phred quality scores using the default MG-RAST pipeline options. Sequences having base calls with phred quality score, Q<15 were filtered out. Sequences that passed primary QC were subjected to secondary QC through MG-RAST's rRNA pipeline under default parameters to remove non-rRNA sequences. Sequences with less than 70% identity to rRNA sequences from M5RNA database were filtered out.

Food type	Method	MG-RAST ID	High-quality reads after primary QC[a]	High-quality reads after secondary QC[b]	Average length (bp)
Fermented bamboo shoot					
	II	4601569.3	6 882	6 878	354
		4601618.3	6 660	6 657	351
		4601557.3	12 097	12 093	350
		4601587.3	12 306	12 285	353
		4601615.3	8 757	8 750	349
		4601593.3	3 800	3 793	352
		4601599.3	13 907	13 896	352
		4601576.3	2 553	2 551	355
	VII				
		4601583.3	7 624	7 615	353
		4601563.3	10 521	10 515	352
		4601598.3	19 043	19 032	350
		4601607.3	17 778	17 752	353
		4601605.3	17 121	17 109	347
		4601624.3	473	472	358
		4601580.3	977	977	356
		4601562.3	2 252	2 251	354

	VIII				
		4601566.3	5 790	5 784	351
		4601596.3	8 473	8 469	350
		4601565.3	8 047	8 038	351
		4601581.3	12 405	12 385	349
		4601579.3	24 848	24 829	348
		4601568.3	2 130	2 125	355
		4601606.3	11 735	11 723	354
		4601597.3	3 352	3 351	356
	AM				
		4601664.3	32 994	32 968	353
		4601635.3	51 777	51 724	352
		4601656.3	20 135	20 114	350
		4601686.3	28 344	28 331	354
		4601684.3	15 107	15 089	351
		4601662.3	25 581	25 560	355
		4601629.3	15 373	15 352	352
		4601654.3	25 565	25 535	353
	DM	4601592.3	15 825	15 812	350
		4601571.3	18 112	18 097	348
		4601590.3	14 562	14 548	349
		4601617.3	13 339	13 326	351
		4601616.3	35 364	35 333	350
		4601595.3	13 717	13 697	354
		4601574.3	8 322	8 317	350
		4601572.3	28 551	28 531	350
Fermented milk					
	II	4601678.3	51 528	51 487	356
		4601695.3	3 115	3 113	356
		4601690.3	1 732	1 730	354
		4601734.3	1 821	1 819	355
		4601709.3	15 122	15 118	356

	4601701.3	5 355	5 353	354
	4601747.3	21 193	21 173	357
	4601697.3	4 577	4 573	355
V				
	4601659.3	6 565	6 556	346
	4601712.3	2 051	2 051	353
	4601706.3	1 631	1 629	355
	4601746.3	4 611	4 603	356
	4601693.3	6 678	6 673	357
	4601719.3	41 492	41 446	355
	4601733.3	12 254	12 243	355
	4601716.3	2 444	2 442	354
VII				
	4601725.3	9 923	9 916	354
	4601749.3	24 460	24 441	355
	4601741.3	2 575	2 574	355
	4601708.3	33 028	33 008	355
	4601729.3	30 269	30 241	354
	4601714.3	31 956	31 939	355
	4601711.3	6 690	6 686	354
	4601704.3	24 964	24 939	355
AM	4601660.3	12 842	12 826	354
	4601670.3	11 886	11 872	355
	4601626.3	18 651	18 628	356
	4601666.3	19 535	19 523	356
	4601645.3	27 187	27 147	354
	4601658.3	33 964	33 938	356
	4601665.3	37 016	36 980	355
	4601675.3	42 559	42 520	356
DM				
	4601594.3	25 138	25 121	354
	4601602.3	5 297	5 293	354

		4601575.3	12 648	12 631	356
		4601614.3	10 262	10 251	354
		4601561.3	50 599	50 557	355
		4601609.3	23 364	23 348	356
		4601610.3	24 701	24 690	355
		4601582.3	20 705	20 682	355
Fermented fish					
	I	4601696.3	5 292	5 288	352
		4601707.3	59 198	59 156	353
		4601691.3	1 070	1 069	358
		4601702.3	53 881	53 841	356
		4601731.3	42 710	42 664	354
	V				
		4601730.3	1 256	1 254	353
		4601739.3	56 460	56 407	355
		4601724.3	1 114	1 112	357
		4601732.3	30 235	30 205	354
		4601703.3	845	845	353
	VII				
		4601713.3	43 116	43 078	352
		4601737.3	1 186	1 184	351
		4601735.3	886	886	354
		4601748.3	44 286	44 257	356
		4601748.3	702	702	358
	AM				
		4601647.3	5 572	5 568	354
		4601640.3	17 992	17 982	357
		4601636.3	35 061	35 027	357
		4601673.3	30 091	30 071	357
		4601622.3	25 048	25 030	356
	DM				

		4601577.3	5 362	5 358	353
		4601556.3	19 081	19 065	354
		4601559.3	25 041	25 030	357
		4601603.3	21 321	21 314	356
		4601585.3	28 612	28 597	357
Fermented soybean					
	I	4601677.3	4 640	4 637	355
		4601657.3	13 176	13 164	356
		4601669.3	36 057	36 036	355
		4601633.3	29 013	29 000	355
		4601682.3	26 075	26 062	356
		4601680.3	32 300	32 283	355
		4601671.3	25 520	25 493	354
		4601672.3	7 148	7 140	353
		4601652.3	6 979	6 976	353
		4601638.3	11 817	11 800	351
	II				
		4601628.3	36 324	36 301	357
		4601644.3	2 980	2 980	354
		4601623.3	56 132	56 094	354
		4601685.3	33 707	33 678	354
		4601630.3	25 409	25 394	355
		4601632.3	12 787	12 767	356
		4601621.3	37 254	37 241	355
		4601620.3	12 160	12 146	353
		4601661.3	34 508	34 469	355
		4601655.3	4 081	4 079	352
	V				
		4601646.3	24 816	24 796	354
		4601627.3	3 011	3 008	354
		4601639.3	31 383	31 359	356
		4601667.3	25 205	25 183	354
		4601649.3	18 865	18 860	356

	4601650.3	21 540	21 528	356
	4601642.3	4 915	4 912	355
	4601641.3	10 562	10 556	351
	4601681.3	33 414	33 395	355
	4601668.3	3 550	3 545	350
AM				
	4601584.3	21 080	21 064	356
	4601589.3	29 868	29 853	355
	4601612.3	22 968	22 948	354
	4601611.3	16 634	16 621	355
	4601588.3	10 087	10 079	353
	4601560.3	8 482	8 473	353
	4601600.3	12 219	12 208	356
	4601567.3	13 012	12 998	355
	4601601.3	13 714	13 698	355
	4601676.3	7 938	7 937	353
DM				355
	4601718.3	9 764	9 760	354
	4601692.3	45 797	45 765	355
	4601717.3	5 100	5 093	356
	4601742.3	2 285	2 280	354
	4601721.3	3 061	3 058	356
	4601687.3	2 916	2 912	355
	4601738.3	5 198	5 196	355
	4601727.3	36 474	36 457	355
	4601740.3	14 506	14 495	356
	4601689.3	14 818	14 806	353
Total		2 781 254	2 778 997	

Table S5. Additional species-level OTUs extracted by each specific method from four food types

Fermented bamboo shoot		
II	VII	VIII
Acinetobacter johnsonii	*Acholeplasma pleciae*	*Acholeplasma modicum*
Acinetobacter schindleri	*Acinetobacter sp. E-10*	*Acinetobacter baumannii*
actinobacterium Aac-25	*Actinomyces georgiae*	*Aerococcus urinaeequi*
Actinomyces urogenitalis	*Actinomyces odontolyticus*	*Alcaligenes faecalis*
Anaerococcus hydrogenalis	*Aeromicrobium marinum*	*Alkaliphilus oremlandii*
Anaerococcus vaginalis	*Aeromonas veronii*	*Alkaliphilus transvaalensis*
Azospirillum irakense	*Anabaena azotica*	*Azospirillum amazonense*
Bacillus badius	*Aneurinibacillus aneurinilyticus*	*Azospirillum lipoferum*
Brevibacillus brevis	*Azospirillum oryzae*	*Bacillus halodurans*
Caenorhabditis remanei	*Azospirillum sp. B510*	*Bacillus mojavensis*
Clostridium innocuum	*Azospirillum zeae*	*Bacillus sp. 2479*
Corynebacterium mastitidis	*Bacillus coagulans*	*Bacillus sp. 2-9-3*
Deinococcus deserti	*Bacillus flexus*	*Bacillus sp. BT97*
Deinococcus ficus	*Bacillus sp. Bt 27*	*Bacillus sp. T03-2A*
Deinococcus radiophilus	*Bacillus sp. SH3*	*Bradyrhizobium sp. STM 2462*
Desulfosporosinus sp. DB	*Bacillus vallismortis*	*Bradyrhizobium sp. STM 2463*
Enterococcus casseliflavus	*Brachybacterium paraconglomeratum*	*Brevundimonas subvibrioides*
Enterococcus durans	*Burkholderia sp. 383*	*Brevundimonas vesicularis*
Enterococcus italicus	*Burkholderia thailandensis*	*Candidatus Solibacter usitatus*
Halomonas variabilis	*Candidatus Odyssella thessalonicensis*	*Chlamydomonas moewusii*
Hymenostylium recurvirostrum	*Caulobacter henricii*	*Clostridium aceticum*
Klebsiella pneumoniae	*Clostridium longisporum*	*Clostridium glycolicum*
Lactobacillus acidifarinae	*Clostridium novyi*	*Clostridium paraputrificum*
Lactobacillus graminis	*Clostridium oceanicum*	*Clostridium sp. MK12*
Lactobacillus hammesii	*Comamonas terrigena*	*Clostridium xylanolyticum*
Lactobacillus jensenii	*Corynebacterium ammoniagenes*	*Dactylosporangium salmoneum*
Lactobacillus letivazi	*Desulfitobacterium hafniense*	*Deinococcus grandis*
Lactobacillus parabrevis	*Desulfitobacterium sp. CR1*	*Deinococcus proteolyticus*
Lactobacillus versmoldensis	*Dyella ginsengisoli*	*Enterococcus aquimarinus*
Leuconostoc carnosum	*Enterococcus faecalis*	*Erythrobacter sp. S14-1*
Macrococcus brunensis	*Enterococcus sulfureus*	*Fructobacillus ficulneus*
Methylobacterium hispanicum	*Fusobacterium nucleatum*	*Geobacillus thermoglucosidasius*
Microlunatus phosphovorus	*Geobacillus jurassicus*	*Haemophilus haemolyticus*
Nesterenkonia halobia	*Geobacillus thermocatenulatus*	*Kocuria rosea*
Nocardioides albus	*Geobacillus thermoleovorans*	*Lactobacillus amylophilus*
Nocardioides aromaticivorans	*Gluconacetobacter sacchari*	*Lactobacillus kefiranofaciens*
Paracoccus solventivorans	*Holdemania filiformis*	*Lactobacillus kunkeei*
Pectobacterium carotovorum	*Kluyvera ascorbata*	*Lactobacillus pentosus*
Pediococcus acidilactici	*Lactobacillus equi*	*Lactobacillus pontis*
Pediococcus claussenii	*Lactobacillus fructivorans*	*Lactobacillus rapi*
Pelomonas saccharophila	*Lactobacillus pantheris*	*Lactobacillus sanfranciscensis*
Peptoniphilus sp. oral taxon 386	*Lactobacillus phage Sal3*	*Lactobacillus ultunensis*

Phalaenopsis aphrodite	*Lactobacillus vaccinostercus*	*Lactococcus garvieae*
Pseudonocardia zijingensis	*Lactococcus piscium*	*Marinococcus halophilus*
Rhodovibrio salinarum	*Leuconostoc gasicomitatum*	*Neisseria flavescens*
Salmonella enterica	*Lysinibacillus fusiformis*	*Peptostreptococcus anaerobius*
Sporomusa ovata	*Lysobacter sp. OC7*	*Pigmentiphaga kullae*
Staphylococcus arlettae	*Macrococcus bovicus*	*Planococcus sp. S5*
Staphylococcus chromogenes	*Megasphaera micronuciformis*	*Polytoma oviforme*
Staphylococcus hominis	*Meiothermus chliarophilus*	*Pseudochlorella pringsheimii*
Staphylococcus succinus	*Methylobacterium fujisawaense*	*Pseudomonas putida*
Streptococcus anginosus	*Methylobacterium persicinum*	*Pseudomonas syringae*
Thermus scotoductus	*Methylobacterium podarium*	*Psychrobacter glacincola*
Tissierella sp. AIP 285.00	*Methylobacterium sp. Hojyo1*	*Rhizobium sp. LXD30*
Tissierella sp. LBN 292	*Nocardioides sp. C190*	*Rhodovulum sp. CP-10*
Tissierella sp. LBN 299	*Nostoc sp. PCC 7423*	*Sarcocaulon vanderietiae*
Truepera radiovictrix	*Opitutus terrae*	*Selenomonas flueggei*
uncultured epsilon proteobacterium	*Pantoea agglomerans*	*Selenomonas lacticifex*
uncultured Ralstonia sp.	*Pediococcus ethanolidurans*	*Selenomonas ruminantium*
Weissella viridescens	*Phegopteris hexagonoptera*	*Selenomonas sputigena*
Wolbachia pipientis	*Pinus merkusii*	*Shewanella putrefaciens*
	Pinus thunbergii	*Staphylococcus devriesei*
	Pisum sativum	*Stichococcus bacillaris*
	Propionibacterium acidipropionici	*Streptococcus suis*
	Prosthecobacter vanneervenii	*Tissierella creatinini*
	Rahnella sp. 9-Pear	*uncultured Bacillus sp.*
	Rhodovulum sulfidophilum	*uncultured Verrucomicrobia bacterium*
	Sedimentibacter hydroxybenzoicus	*Veillonella dispar*
	Staphylococcus microti	*Verrucomicrobiaceae bacterium CHC12*
	Staphylococcus saprophyticus	*Verrucomicrobium spinosum*
	Stenotrophomonas rhizophila	*Verticillium dahliae*
	Streptococcus pyogenes	*Weissella minor*
	Streptococcus salivarius	
	Terrabacter sp. YK3	
	Tissierella praeacuta	
	Trifolium repens	
	uncultured marine bacterium	
	uncultured Methylobacterium sp.	
	uncultured organism	
	uncultured proteobacterium	
	uncultured rumen bacterium	
	Ureibacillus thermosphaericus	
	Verrucomicrobiaceae bacterium ONA9	
	Xanthomonas campestris	
Fermented milk		
II	V	VII
Abiotrophia defectiva	*Acholeplasma axanthum*	*Acetobacter estunensis*
Acetobacter lovaniensis	*Acinetobacter johnsonii*	*Acidithiobacillus thiooxidans*

Acetobacter syzygii
Acidovorax valerianellae
Aeromonas veronii
Aneurinibacillus thermoaerophilus
Bacillus caldolyticus
Bacillus fordii
Bacillus litoralis
Bacillus sp. BT97
Bacillus sp. GB02-31
Bacillus sp. KHg1
Bacillus sp. N2a
Bacillus thuringiensis
Brevundimonas bullata
Caloramator fervidus
Carnobacterium sp. St2
Citrobacter sedlakii
Citrus clementina
Clostridium saccharobutylicum
Comamonas aquatica
Corynebacterium flavescens
Deinococcus murrayi
Desulfitobacterium hafniense
Enterococcus malodoratus
Gluconacetobacter diazotrophicus
Granulicatella elegans
Halobacillus dabanensis
Janibacter sp. BY48
Lactobacillus amylotrophicus
Lactobacillus cacaonum
Lactobacillus equi
Lactobacillus iners
Lactobacillus mucosae
Lactobacillus panis
Lactobacillus spicheri
Leuconostoc fallax
Macrococcus caseolyticus
Mannheimia sp. NCTC 11313
Mannheimia varigena
Marinococcus halophilus
Methylobacterium hispanicum
Methylobacterium sp. CBMB38
Micrococcus luteus
Morganella morganii
Nesterenkonia halobia
Nocardioides albus

Agrobacterium larrymoorei
Bacillus coagulans
Bacillus niacini
Bacillus sp. JAMB-204
Brevibacillus borstelensis
Clostridium formicaceticum
Corynebacterium accolens
Corynebacterium variabile
Deinococcus radiophilus
Enterococcus cecorum
Enterococcus silesiacus
Enterococcus sp. DF14
Enterocytozoon bieneusi
Eubacterium hallii
Frankia sp.
Geobacillus thermoglucosidasius
Geobacillus thermoleovorans
Geobacillus vulcani
Kluyvera ascorbata
Lactobacillus alimentarius
Lactobacillus kalixensis
Lactobacillus ruminis
Lactobacillus sp.
Lactobacillus vaccinostercus
Leuconostoc palmae
Marinobacter sp. HS7
Methylobacterium aquaticum
Natronobacillus azotifigens
Nocardioides sp. CF8
Oceanobacillus picturae
Ochrobactrum grignonense
Opitutus sp. VeGlc2
Pimelobacter simplex
Polypodium aureum
Prosthecobacter vanneervenii
Pseudomonas sp. PTB2093
Pullulanibacillus naganoensis
Shewanella sp. ANA-3
Shewanella sp. MR-7
Staphylococcus pseudolugdunensis
Streptococcus pluranimalium
Streptomyces tricolor
Tissierella sp. LBN 292
Trichococcus flocculiformis
Vagococcus salmoninarum

Aerococcus sanguinicola
Aerococcus urinaeequi
Aeromonas schubertii
Anoxybacillus flavithermus
Bacillus badius
Bacillus mojavensis
Bacillus sp. JL-39
Bacillus sp. SH3
Bradyrhizobium japonicum
Brevundimonas subvibrioides
Clostridium butyricum
Clostridium difficile
Clostridium histolyticum
Clostridium oceanicum
Clostridium sardiniense
Coptotermes formosanus
Corynebacterium glucuronolyticum
Corynebacterium macginleyi
Cryobacterium psychrophilum
Deinococcus ficus
Enterobacter sp. SA-A5-114
Enterococcus hirae
Erwinia amylovora
Fructobacillus ficulneus
Halobacillus karajensis
Klebsiella sp. MNFG_801b
Kurthia sibirica
Lactobacillus hamsteri
Lactobacillus murinus
Lactobacillus psittaci
Lactobacillus vaginalis
Leclercia adecarboxylata
Listeria grayi
Methylobacterium podarium
Microbacterium chocolatum
Microlunatus phosphovorus
Morganella psychrotolerans
Nesterenkonia aethiopica
Nocardioides sp. CMU5
Ochrobactrum sp. mp-3
Opitutus terrae
Peptoniphilus harei
Propionibacterium granulosum
Proteus mirabilis
Pseudomonas pseudoalcaligenes

Nocardioides sp. MTD22	*Vagococcus teuberi*	*Pseudomonas putida*
Oceanobacillus chironomi		*Psychrobacter glacincola*
Paenibacillus lactis		*Rahnella aquatilis*
Paenibacillus sp. SH-55		*Rahnella sp. 9-Pear*
Paralactobacillus selangorensis		*Saccharopolyspora taberi*
Pediococcus pentosaceus		*Selenomonas lacticifex*
Planococcus sp. L4		*Serratia fonticola*
Pseudomonas aeruginosa		*Staphylococcus auricularis*
Pseudomonas brenneri		*Staphylococcus hominis*
Pseudomonas marincola		*Streptococcus caballi*
Pseudomonas rhodesiae		*Streptococcus infantarius*
Pseudomonas syringae		*Streptococcus iniae*
Rickettsiella grylli		*Streptococcus pneumoniae*
Sporosarcina pasteurii		*Thermus igniterrae*
Staphylococcus massiliensis		*Truepera radiovictrix*
Staphylococcus pasteuri		*uncultured alpha proteobacterium*
Streptococcus minor		*uncultured soil bacterium*
Streptococcus parauberis		*Vagococcus carniphilus*
Streptococcus uberis		*Yersinia pseudotuberculosis*
Thermus brockianus		
Yersinia pestis		
Fermented fish		
I	V	VII
Acetobacter pomorum	*Arthrobacter arilaitensis*	*Aerococcus sanguinicola*
Alkaliphilus transvaalensis	*Arthrobacter pascens*	*Amycolatopsis albidoflavus*
Anaerococcus lactolyticus	*Bacillus fordii*	*Bacillus sp. GB02-14C*
Anaerococcus prevotii	*Bosea vestrisii*	*Bacillus sp. NB-6*
Aquaspirillum serpens	*Brachybacterium tyrofermentans*	*Bacillus thuringiensis*
Arthrobacter keyseri	*Bradyrhizobium sp. STM 2462*	*Bavariicoccus seileri*
Atopobacter phocae	*Bradyrhizobium sp. STM 2463*	*Clostridium histolyticum*
Bacillus aquimaris	*Brevibacillus laterosporus*	*Clostridium limosum*
Bacillus firmus	*Caenorhabditis remanei*	*Clostridium paradoxum*
Bacillus hwajinpoensis	*Clostridium beijerinckii*	*Clostridium xylanolyticum*
Bacillus megaterium	*Clostridium longisporum*	*Coptotermes formosanus*
Bacillus sp. BT97	*Clostridium sp. CA6*	*Corynebacterium glucuronolyticum*
Bacillus sp. HM06-02	*Clostridium taeniosporum*	*Dietzia maris*
Bacillus sp. JAMB-204	*Corynebacterium cyclohexanicum*	*Enterococcus faecalis*
Bacillus sporothermodurans	*Desulfitobacterium hafniense*	*Enterococcus gallinarum*
Bacteroides cellulosolvens	*Enterococcus avium*	*Finegoldia magna*
Brachybacterium paraconglomeratum	*Enterococcus saccharolyticus*	*Geobacillus kaustophilus*
Bradyrhizobium japonicum	*Fructobacillus ficulneus*	*Kocuria rhizophila*
Bradyrhizobium sp. ISLU207	*Fructobacillus pseudoficulneus*	*Kutzneria viridogrisea*
Brevundimonas bullata	*Geobacillus thermoleovorans*	*Lactobacillus curvatus*
Brevundimonas subvibrioides	*Granulicatella adiacens*	*Lactobacillus saerimneri*
Campylobacter hominis	*Halomonas aquamarina*	*Mechercharimyces mesophilus*
Candidatus Koribacter versatilis	*Lactobacillus backi*	*Morganella morganii*

Caulobacter henricii
Chlamydophila abortus
Clostridium aceticum
Clostridium carnis
Clostridium cellulolyticum
Clostridium hiranonis
Clostridium kluyveri
Clostridium saccharobutylicum
Clostridium saccharoperbutylacetonicum
Clostridium sardiniense
Clostridium tertium
Corynebacterium aurimucosum
Corynebacterium bovis
Corynebacterium macginleyi
Deinococcus deserti
Deinococcus geothermalis
Deinococcus murrayi
Deinococcus proteolyticus
Deinococcus radiodurans
Desulfosporosinus sp. DB
Enterococcus thailandicus
Eubacterium biforme
Frankia sp. MgI5
Fructobacillus fructosus
Geobacillus sp. T45
Geobacillus sp. Y412MC61
Geobacillus thermocatenulatus
Halobacillus dabanensis
Ignatzschineria indica
Kribbella catacumbae
Lactobacillus amylotrophicus
Lactobacillus letivazi
Lactobacillus pentosus
Lactobacillus vini
Legionella anisa
Leuconostoc inhae
Listeria seeligeri
Macrococcus brunensis
Marinococcus halophilus
Megasphaera micronuciformis
Methylobacterium hispanicum
Mitsuokella multacida
Nesterenkonia sp. AN1
Nocardioides aromaticivorans
Nocardioides sp. AN3
Oxobacter pfennigii
Paenibacillus anaericanus
Paenibacillus lentimorbus
Paenibacillus polymyxa

Lactobacillus cacaonum
Lactobacillus fuchuensis
Lactobacillus graminis
Lactobacillus helveticus
Lactobacillus kalixensis
Lactobacillus kefiri
Lactobacillus malefermentans
Lactobacillus mindensis
Lactobacillus salivarius
Lactobacillus vaccinostercus
Lactobacillus versmoldensis
Legionella lytica
Leucobacter komagatae
Methylobacterium jeotgali
Methylobacterium persicinum
Paenibacillus amylolyticus
Protochlamydia naegleriophila
Ralstonia solanacearum
Raoultella terrigena
Rhodopseudomonas palustris
Tissierella sp. AIP 285.00
uncultured beta proteobacterium
uncultured Enterobacter sp.
Vagococcus salmoninarum
Vibrio fortis
Wohlfahrtiimonas chitiniclastica

Oceanobacillus chironomi
Paenibacillus sp. JDR-2
Proteus penneri
Proteus vulgaris
Pseudomonas putida
Psychrobacter fozii
Rahnella aquatilis
Rahnella sp. 9-Pear
Rhodocista pekingensis
rumen bacterium NK3B85
Sporosarcina saromensis
Staphylococcus lugdunensis
Staphylococcus vitulinus
Terribacillus halophilus
Weissella minor
Weissella viridescens

Paenibacillus sp. C7		
Paracoccus sp. R-24652		
Parvimonas micra		
Pediococcus acidilactici		
Peptoniphilus asaccharolyticus		
Pseudomonas brenneri		
Pseudomonas chlororaphis		
Pseudomonas pseudoalcaligenes		
Rhizobium tropici		
Shewanella putrefaciens		
Shewanella sp. MR-4		
Sporanaerobacter acetigenes		
Sporomusa ovata		
Sporosarcina pasteurii		
Staphylococcus auricularis		
Staphylococcus caprae		
Staphylococcus devriesei		
Staphylococcus pseudolugdunensis		
Staphylococcus xylosus		
Streptococcus parauberis		
Streptococcus porci		
Streptococcus sanguinis		
Syntrophobacter wolinii		
Terriglobus saanensis		
Thauera terpenica		
Tissierella praeacuta		
Tissierella sp. LBN 291		
Truepera radiovictrix		
uncultured delta proteobacterium		
Veillonella ratti		
Verrucomicrobiaceae bacterium ONA9		
Virgibacillus carmonensis		
Weissella confusa		
Yersinia pseudotuberculosis		

Fermented soybean		
I	II	V
Acinetobacter sp. A1PC16	*Acetobacter estunensis*	*Acidobacterium capsulatum*
Actinomyces odontolyticus	*Acidovorax facilis*	*Acinetobacter junii*
Aerococcus urinae	*Acinetobacter sp. AD513A*	*actinobacterium Aac-25*
Agrobacterium vitis	*Alicyclobacillus acidoterrestris*	*Aerococcus sanguinicola*
Atopobacter phocae	*Amycolatopsis coloradensis*	*Aeromonas veronii*
Bacillus atrophaeus	*Anoxybacillus kestanbolensis*	*Bacillus flexus*
Bacillus caldolyticus	*Azospirillum irakense*	*Bacillus hwajinpoensis*
Bacillus fastidiosus	*Azospirillum sp. B510*	*Bacillus simplex*
Bacillus safensis	*Azospirillum sp. BV-s*	*Bacillus sp. MB-11*
Bavariicoccus seileri	*Bacillus anthracis*	*Bacillus sp. SG-1*

Bifidobacterium animalis
Bifidobacterium longum
Brevibacillus brevis
Brevibacillus laterosporus
Brucella abortus
Brucella melitensis
Brucella suis
Chryseobacterium formosense
Clostridium cellobioparum
Clostridium disporicum
Clostridium sphenoides
Clostridium tetani
Corynebacterium singulare
Desulfosporosinus sp. DB
Enterococcus asini
Enterococcus avium
Enterococcus italicus
Enterococcus saccharolyticus
Enterococcus thailandicus
Eragrostis curvula
Erwinia papayae
Erwinia persicina
Geobacillus sp. G11MC16
Geobacillus sp. T45
Halobacillus trueperi
Halomonas aquamarina
Halomonas salina
Ignatzschineria ureiclastica
Klebsiella variicola
Lachnospira multipara
Lachnospiraceae bacterium A4
Lactobacillus amylolyticus
Lactobacillus curvatus
Lactobacillus ingluviei
Lactobacillus oris
Lactobacillus pentosus
Lactococcus sp. JIP 26-01
Methylobacterium fujisawaense
Paenibacillus sp. HanTHS1
Paenibacillus sp. KSM-N440
Paracoccus sp. R-24652
Planomicrobium okeanokoites
Pseudomonas nitroreducens
Psychrobacter frigidicola
Rhizobium etli
Rhodovulum sulfidophilum
Ruminococcus bromii
Sedimentibacter hydroxybenzoicus

Bacillus beijingensis
Bacillus cytotoxicus
Bacillus halmapalus
Bacillus horikoshii
Bacillus niacini
Bacillus sp. 2-9-3
Bacillus sp. HH-01
Bacillus sp. MD2
Bacillus sp. NB-6
Bacillus sp. PS1
Bacillus sp. RH219
Bacillus sp. T107
Bradyrhizobium sp. SEMIA 6186
Bradyrhizobium yuanmingense
Brevibacillus parabrevis
Caulobacter fusiformis
Clostridium cylindrosporum
Clostridium formicaceticum
Clostridium longisporum
Corynebacterium diphtheriae
Corynebacterium flavescens
Corynebacterium simulans
Draba nemorosa
Enterobacter aerogenes
Enterobacter amnigenus
Enterococcus gallinarum
Exiguobacterium sibiricum
Fructobacillus ficulneus
Gluconobacter sp. aP78
Gluconobacter thailandicus
Guizotia abyssinica
Halobacillus litoralis
Halomonas campaniensis
Klebsiella sp. MNFG_801b
Kluyvera ascorbata
Kurthia zopfii
Lactobacillus fructivorans
Lactobacillus saerimneri
Lactobacillus sp. DCY51
Leptolyngbya foveolarum
Leptolyngbya sp. MMG-1
Leuconostoc citreum
Leuconostoc palmae
Macrococcus brunensis
Mannheimia varigena
Meiothermus chliarophilus
Microbacterium arabinogalactanolyticum
Microbacterium esteraromaticum

Bradyrhizobium japonicum
Brevibacterium casei
Brevibacterium mcbrellneri
Brevundimonas intermedia
Brevundimonas subvibrioides
Caulobacter henricii
Clavibacter michiganensis
Clostridium sardiniense
Comamonas testosteroni
Deinococcus grandis
Enterobacter asburiae
Enterobacter turicensis
Enterococcus malodoratus
Enterococcus mundtii
Eubacterium siraeum
Klebsiella pneumoniae
Kutzneria viridogrisea
Lactobacillus equi
Lactobacillus graminis
Lactobacillus iners
Lactobacillus manihotivorans
Lactobacillus pontis
Legionella yabuuchiae
Leuconostoc inhae
Megasphaera micronuciformis
Methylobacterium hispanicum
Methylobacterium populi
Methylobacterium sp. Hojyo1
Microbacterium oxydans
Nocardioides aromaticivorans
Paenibacillus ehimensis
Paenibacillus lautus
Pantoea ananatis
Providencia stuartii
Pseudomonas migulae
Rheinheimera sp. Chandigarh
Sanguibacter keddieii
Sarcina ventriculi
Shewanella algae
Sporomusa ovata
Staphylococcus carnosus
Streptococcus minor
Streptococcus suis
Streptococcus vestibularis
Streptomyces caeruleus
Thermincola potens
Trichormus azollae
uncultured Klebsiella sp.

Shewanella halifaxensis	*Microbacterium laevaniformans*	*uncultured prokaryote*
Shigella dysenteriae	*Micrococcus lylae*	*Yersinia pseudotuberculosis*
Staphylococcus auricularis	*Nesterenkonia halobia*	
Staphylococcus gallinarum	*Nesterenkonia sp. 10004*	
Staphylococcus hominis	*Paenibacillus thiaminolyticus*	
Staphylococcus succinus	*Peptoniphilus harei*	
Stenotrophomonas rhizophila	*Phalaenopsis aphrodite*	
Terrabacter tumescens	*Providencia rettgeri*	
Tsukamurella pulmonis	*Pseudomonas aeruginosa*	
Veillonella parvula	*Pseudomonas alcaligenes*	
Virgibacillus marismortui	*Psychrobacter immobilis*	
Zimmermannella faecalis	*Rahnella sp. 9-Pear*	
	Ralstonia solanacearum	
	Rhodococcus pyridinivorans	
	Rothia nasimurium	
	Serinicoccus marinus	
	Shewanella sp. ANA-3	
	Shewanella sp. MR-7	
	Shigella flexneri	
	Spathiphyllum wallisii	
	Sporanaerobacter acetigenes	
	Staphylococcus devriesei	
	Staphylococcus haemolyticus	
	Staphylococcus hyicus	
	Staphylococcus xylosus	
	Streptococcus gallolyticus	
	Thermoactinomyces vulgaris	
	uncultured Bacillus sp.	
	uncultured organism	
	uncultured rumen bacterium	
	Verrucomicrobiaceae bacterium CHC12	
	Vitis vinifera	
	Weissella koreensis	
	Weissella viridescens	
	Xanthomonas campestris	
	Zymobacter palmae	

Table S6. **Mean relative abundance of genus-level eubacterial OTUs recovered by different extraction methods (I, II, V, VII, and VIII) and significant differences of the means in three food types**. Data is derived from the replicate analysis of eight independent experiments (fermented bamboo and fermented milk) and ten independent experiments (fermented soybean) for each method. No significant difference in the recovery of genus-level eubacterial OTUs in fermented fish was recorded. *P*-values were calculated using Student's two-tailed paired *t*-test, $P<0.05$ is indicated in bold.

	**Mean relative abundance (%)**			***P*-value**		
Fermented bamboo	II	VII	VIII	II vs VII	II vs VIII	VII vs VIII
Lactobacillus	70.61 ± 6.06	42.65 ± 4.86	76.02 ± 6.82	**0.008**	0.573	**0.002**
Bacillus	4.50 ± 0.91	26.59 ± 5.75	3.21 ± 0.81	**0.007**	0.402	**0.003**
Propionibacterium	0.42 ± 0.23	0.44 ± 0.11	0.04 ± 0.01	0.905	0.141	**0.006**
Shewanella	0.39 ± 0.11	0.40 ± 0.15	0.05 ± 0.01	0.936	**0.015**	0.050
Methylobacterium	0.08 ± 0.01	0.15 ± 0.04	0.05 ± 0.04	0.217	0.130	**0.038**
Fermented milk	II	V	VII	II vs V	II vs VII	V vs VII
Lactobacillus	46.858 ± 9.18	24.367± 10.60	29.307 ± 5.98	0.063	**0.019**	0.613
Gluconobacter	0.66 ± 0.10	20.46 ± 8.38	13.59 ± 4.12	**0.022**	0.066	0.312
Thermus	1.27 ± 0.61	0.03 ± 0.01	0.38 ± 0.35	**0.041**	**0.028**	0.243
Fermented soybean	I	II	V	I vs II	I vs V	II vs V
Lactobacillus	5.89 ± 2.56	10.22 ± 3.85	5.77 ± 2.55	**0.034**	0.963	0.156
Carnobacterium	0.26 ± 0.08	2.46 ± 1.50	0.05 ± .02	0.181	**0.038**	0.144

Table S7. **Mean relative abundance of species-level eubacterial OTUs recovered by different extraction methods (I, II, V, VII and VIII) and significant differences of the means in four food types.** Data is derived from the replicate analysis of eight independent experiments (fermented bamboo and fermented milk), ten independent experiments (fermented soybean) and five independent experiments (fermented fish). *P*-values were calculated using Student's two-tailed paired *t*-test, *P*<0.05 is indicated in bold. For fish samples, *P*-values were calculated using Student's one-tailed paired *t*-test due to high variation between samples.

	**Mean relative abundance (%)**			***P*-value**		
Fermented bamboo shoot	II	VII	VIII	II vs VII	II vs VIII	VII vs VIII
Lactobacillus acetotolerans	53.85 ± 7.01	23.59 ± 5.38	64.23 ± 6.76	**0.007**	0.307	**0.001**
Bacillus subtilis	2.292 ± 1.00	18.54 ± 5.16	2.205 ± 0.86	**0.014**	0.947	**0.008**
Lactobacillus zymae	0.871 ± 0.95	4.097 ± 2.00	3.700 ± 1.73	**0.036**	0.074	0.810
Lactobacillus acidipiscis	0.728 ± 0.37	1.049 ± 0.37	0.707 ± 0.44	0.563	0.973	**0.041**
Lactobacillus spicheri	3.218 ± 1.02	0.962 ± 0.53	0.971 ± 0.78	0.056	**0.011**	0.993
Lactobacillus delbrueckii	0.835 ± 0.25	1.773 ± 0.37	0.700 ± 0.12	0.063	0.511	**0.030**
Propionibacterium acnes	0.418 ± 0.26	0.388 ± 0.08	0.040 ± 0.01	0.882	0.138	**0.003**
Lactobacillus secaliphilus	0.795 ± 0.27	0.411 ± 0.30	0.041 ± 0.02	0.235	**0.017**	0.269
Bacillus circulans	0.220 ± 0.05	0.298 ± 0.09	0.053 ± 0.02	0.467	0.192	**0.045**
Shewanella haliotis	0.388 ± 0.11	0.403 ± 0.15	0.041 ± 0.02	0.941	**0.012**	0.054
Leuconostoc pseudomesenteroides	0.165 ± 0.04	0.169 ± 0.10	0.030 ± 0.02	0.976	**0.049**	0.178
Fermented milk	II	V	VII	II vs V	II vs VII	V vs VII
Lactobacillus delbrueckii	45.184 ± 9.31	22.10 ± 10.99	28.08 ± 5.85	0.076	**0.032**	0.561
Leuconostoc sp.	7.003 ± 1.28	8.447 ± 5.94	18.881 ± 9.41	0.818	0.247	**0.037**
Bacillus circulans	0.269 ± 0.15	1.158 ± 0.34	0.205 ± 0.13	**0.031**	0.781	**0.019**
Clostridium bifermentans	0.452 ± 0.09	0.378 ± 0.12	0.232 ± 0.02	0.613	**0.046**	0.232
Erysipelothrix rhusiopathiae	0.005 ± 0.00	0.00	0.004 ± 0.00	0.174	0.814	**0.035**
Fermented soybean	I	II	V	I vs II	I vs V	II vs V
Vagococcus fluvialis	1.025 ± 0.98	0.024 ± 0.01	0.076 ± 0.03	0.286	0.308	**0.035**
Clostridium chauvoei	0.021 ± 0.01	0.012 ± 0.00	0.010 ± 0.00	0.106	**0.032**	0.355
Tetragenococcus muriaticus	0.090 ± 0.02	0.113 ± 0.03	0.075 ± 0.02	0.466	0.538	**0.036**

Fermented fish	I	V	VII	I vs V	I vs VII	V vs VII
Clostridium bifermentans	33.303 ± 14.05	4.102 ± 1.94	15.998 ± 10.66	**0.042**	0.125	0.127
Bacillus subtilis	0.680 ± 0.27	2.100 ± 0.68	1.890 ± 0.94	**0.049**	0.153	0.391
Staphylococcus cohnii	4.152 ± 1.47	1.686 ± 0.68	0.917 ± 0.42	0.075	**0.026**	0.215
Leuconostoc mesenteroides	0.426 ± 0.15	0.579 ± 0.25	1.091 ± 0.36	0.320	**0.036**	0.069
Bacillus circulans	0.070 ± 0.06	0.380 ± 0.18	0.544 ± 0.37	**0.039**	0.142	0.374
Acetobacter pasteurianus	0.130 ± 0.06	2.314 ± 2.24	0.017 ± 0.01	0.189	**0.045**	0.181
Lactobacillus sakei	0.135 ± 0.13	0.073 ± 0.05	0.210 ± 0.16	0.285	**0.049**	0.180

Table S8. Summary of SIMPER analysis showing species-level OTUs that contribute to 50% variability in the microbial community structure for each food type recovered by different methods (I, II, V, VII and VIII). Analysis was performed using Bray-Curtis dissimilarity matrix derived from the pooled relative abundance of species-level OTUs across the three different extraction methods for each food type. Relative abundances are expressed as mean for the replicate of eight independent experiments (fermented bamboo and fermented milk), five independent experiments (fermented fish) and ten independent experiments (fermented soybean).

	**Average Bray Curtis dissimilarity**	**Dissimilarity contribution (%)**	**Mean relative abundance (%)**		
Fermented bamboo shoot			II	VII	VIII
Lactobacillus acetotolerans	16.02	29.12	53.9	23.6	64.2
Bacillus subtilis	5.9	10.72	2.29	18.5	2.21
Phenylobacterium sp.	4.28	7.78	4.22	11.3	5.84
Bacillus pumilus	2.41	4.38	0.70	6.69	0.46
Fermented milk			II	V	VII
Lactobacillus delbrueckii	15.58	22.32	45.2	22.1	28.1
Leuconostoc mesenteroides	9.21	13.2	4.47	22.1	11.9
Leuconostoc sp.	7.45	10.68	7	8.45	18.9
Gluconobacter frateurii	6.99	10.03	0.28	16.4	8.42
Fermented fish			I	V	VII
Clostridium bifermentans	13.11	17.75	33.3	4.1	16
Virgibacillus halophilus	6.84	9.27	9.67	6.17	10.7

Lactobacillus acidipiscis	6.84	9.26	13.2	17.6	10.7
Lactobacillus delbrueckii	5.36	7.26	2.14	10.1	10.2
Lactobacillus rennin	2.70	3.65	0.26	7.87	0.48
Clostridium botulinum	2.51	3.41	3.44	4.28	2.48
Fermented soybean			I	II	V
Bacillus subtilis	12.62	18.96	32.1	28.3	26.6
Atopostipes suicloacalis	5.49	8.25	3.08	4.95	13.1
Atopostipes sp.	4.33	6.50	10.8	3.36	4.79
Bacillus circulans	4.05	6.09	7.72	6.9	7.04
Aerococcus viridians	3.62	5.45	3.47	5.53	5.7
Bacillus pumilus	3.28	4.93	4.93	5.17	5.58

Table S9. Sequence diversity and library coverage estimations of Illumina MiSeq sequencing.

			**Chao1**				
Fermented bamboo shoot							
Group1	Group2	Group1 mean	Group1 SEM	Group2 mean	Group2 SEM	t stat	*P* value
AM	II	97.51	14.38	74.78	8.46	4.09	0.00
AM	VIII	97.51	14.38	74.66	9.71	3.80	0.00
AM	VII	97.51	14.38	78.96	8.61	3.32	0.00
II	DM	74.78	8.46	87.33	10.81	-2.68	0.02
VIII	DM	74.66	9.71	87.33	10.81	-2.46	0.03
VII	DM	78.96	8.61	87.33	10.81	-1.77	0.09
AM	DM	97.51	14.38	87.33	10.81	1.64	0.12
II	VII	74.78	8.46	78.96	8.61	-1.04	0.31
VIII	VII	74.66	9.71	78.96	8.61	-0.97	0.35
VIII	II	74.66	9.71	74.78	8.46	-0.03	0.98
Fermented milk							
Group1	Group2	Group1 mean	Group1 SEM	Group2 mean	Group2 SEM	t stat	*P* value
II	AM	61.60	10.19	81.50	6.87	-4.85	0.00
AM	VII	81.50	6.87	62.20	8.69	5.22	0.00
AM	V	81.50	6.87	58.95	17.29	3.64	0.00
DM	AM	69.98	4.94	81.50	6.87	-3.76	0.00
DM	VII	69.98	4.94	62.20	8.69	2.13	0.05
II	DM	61.60	10.19	69.98	4.94	-2.01	0.06
DM	V	69.98	4.94	58.95	17.29	1.65	0.12
VII	V	62.20	8.69	58.95	17.29	0.50	0.64
II	V	61.60	10.19	58.95	17.29	0.40	0.71
II	VII	61.60	10.19	62.20	8.69	-0.13	0.90

Fermented fish							
Group1	Group2	Group1 mean	Group1 SEM	Group2 mean	Group2 SEM	t stat	*P* value
AM	VII	83.11	17.72	60.25	17.39	2.60	0.02
AM	V	83.11	17.72	66.55	9.70	2.21	0.04
I	AM	67.86	11.17	83.11	17.72	-1.97	0.07
DM	AM	69.93	26.56	83.11	17.72	-1.17	0.26
I	VII	67.86	11.17	60.25	17.39	0.99	0.33
DM	VII	69.93	26.56	60.25	17.39	0.86	0.41
VII	V	60.25	17.39	66.55	9.70	-0.85	0.42
DM	V	69.93	26.56	66.55	9.70	0.32	0.77
I	V	67.86	11.17	66.55	9.70	0.23	0.81
I	DM	67.86	11.17	69.93	26.56	-0.19	0.87

Fermented soybean							
Group1	Group2	Group1 mean	Group1 SEM	Group2 mean	Group2 SEM	t stat	*P* value
II	AM	122.16	15.49	103.69	13.77	2.67	0.01
I	AM	124.92	23.55	103.69	13.77	2.33	0.04
II	V	122.16	15.49	109.39	13.99	1.83	0.08
I	V	124.92	23.55	109.39	13.99	1.70	0.11
DM	AM	114.22	31.04	103.69	13.77	0.93	0.38
AM	V	103.69	13.77	109.39	13.99	-0.87	0.39
I	DM	124.92	23.55	114.22	31.04	0.82	0.42
II	DM	122.16	15.49	114.22	31.04	0.69	0.50
DM	V	114.22	31.04	109.39	13.99	0.43	0.68
II	I	122.16	15.49	124.92	23.55	-0.29	0.78

**Alpha diversity**							
Fermented bamboo shoot							
Group1	Group2	Group1 mean	Group1 SEM	Group2 mean	Group2 SEM	t stat	*P* value
AM	VIII	15.07	2.01	11.08	1.15	4.95	0.00
AM	II	15.07	2.01	12.02	1.27	3.84	0.00
VIII	DM	11.08	1.15	13.59	2.26	-2.81	0.01
AM	VII	15.07	2.01	12.85	2.49	2.08	0.05
VIII	VII	11.08	1.15	12.85	2.49	-1.85	0.08
II	DM	12.02	1.27	13.59	2.26	-1.79	0.09
VIII	II	11.08	1.15	12.02	1.27	-1.61	0.13
AM	DM	15.07	2.01	13.59	2.26	1.43	0.17
II	VII	12.02	1.27	12.85	2.49	-0.89	0.40
VII	DM	12.85	2.49	13.59	2.26	-0.64	0.53
Fermented milk							
Group1	Group2	Group1 mean	Group1 SEM	Group2 mean	Group2 SEM	t stat	*P* value
AM	VII	10.12	0.95	7.43	0.80	6.51	0.00
DM	AM	7.98	0.75	10.12	0.95	-4.91	0.00
II	AM	8.02	1.28	10.12	0.95	-3.95	0.00
AM	V	10.12	0.95	7.48	2.20	3.31	0.00
DM	VII	7.98	0.75	7.43	0.80	1.41	0.18
II	VII	8.02	1.28	7.43	0.80	1.16	0.26
II	V	8.02	1.28	7.48	2.20	0.63	0.56
DM	V	7.98	0.75	7.48	2.20	0.58	0.61
II	DM	8.02	1.28	7.98	0.75	0.06	0.95
VII	V	7.43	0.80	7.48	2.20	-0.07	0.96

Fermented fish							
Group1	Group2	Group1 mean	Group1 SEM	Group2 mean	Group2 SEM	t stat	*P* value
DM	AM	9.21	5.31	12.05	4.75	-1.13	0.28
DM	V	9.21	5.31	11.40	3.99	-0.90	0.39
AM	VII	12.05	4.75	10.32	3.10	0.86	0.40
I	DM	11.10	4.65	9.21	5.31	0.73	0.47
VII	V	10.32	3.10	11.40	3.99	-0.59	0.55
DM	VII	9.21	5.31	10.32	3.10	-0.51	0.64
I	AM	11.10	4.65	12.05	4.75	-0.39	0.70
I	VII	11.10	4.65	10.32	3.10	0.38	0.71
AM	V	12.05	4.75	11.40	3.99	0.28	0.78
I	V	11.10	4.65	11.40	3.99	-0.13	0.90

Fermented soybean							
Group1	Group2	Group1 mean	Group1 SEM	Group2 mean	Group2 SEM	t stat	*P* value
II	AM	15.76	2.28	12.53	2.03	3.17	0.01
I	AM	15.67	3.10	12.53	2.03	2.54	0.02
AM	V	12.53	2.03	14.62	2.95	-1.76	0.10
DM	AM	15.12	4.46	12.53	2.03	1.59	0.14
II	V	15.76	2.28	14.62	2.95	0.91	0.37
I	V	15.67	3.10	14.62	2.95	0.73	0.47
II	DM	15.76	2.28	15.12	4.46	0.38	0.70
I	DM	15.67	3.10	15.12	4.46	0.30	0.77
DM	V	15.12	4.46	14.62	2.95	0.28	0.77
II	I	15.76	2.28	15.67	3.10	0.07	0.94

Shannon diversity

Fermented bamboo shoot

Group1	Group2	Group1 mean	Group1 SEM	Group2 mean	Group2 SEM	t stat	*P* value
VIII	DM	2.22	0.61	3.19	0.54	-3.35	0.01
AM	VIII	3.17	0.68	2.22	0.61	3.01	0.01
VIII	VII	2.22	0.61	3.15	0.92	-2.42	0.03
VIII	II	2.22	0.61	2.88	0.69	-2.07	0.05
II	DM	2.88	0.69	3.19	0.54	-1.02	0.33
AM	II	3.17	0.68	2.88	0.69	0.90	0.38
II	VII	2.88	0.69	3.15	0.92	-0.70	0.50
VII	DM	3.15	0.92	3.19	0.54	-0.11	0.92
AM	DM	3.17	0.68	3.19	0.54	-0.07	0.95
AM	VII	3.17	0.68	3.15	0.92	0.06	0.96

Fermented milk

Group1	Group2	Group1 mean	Group1 SEM	Group2 mean	Group2 SEM	t stat	*P* value
II	AM	2.38	0.57	2.83	0.29	-2.10	0.05
AM	V	2.83	0.29	2.42	0.59	1.87	0.08
DM	AM	2.57	0.36	2.83	0.29	-1.57	0.13
AM	VII	2.83	0.29	2.55	0.50	1.46	0.16
II	DM	2.38	0.57	2.57	0.36	-0.79	0.44
II	VII	2.38	0.57	2.55	0.50	-0.66	0.52
DM	V	2.57	0.36	2.42	0.59	0.61	0.54
VII	V	2.55	0.50	2.42	0.59	0.50	0.63
II	V	2.38	0.57	2.42	0.59	-0.14	0.88
DM	VII	2.57	0.36	2.55	0.50	0.12	0.92

Fermented fish							
Group1	Group2	Group1 mean	Group1 SEM	Group2 mean	Group2 SEM	t stat	*P* value
DM	V	2.80	0.67	3.61	0.78	-2.17	0.04
DM	VII	2.80	0.67	3.47	0.65	-2.01	0.06
I	DM	3.47	0.97	2.80	0.67	1.57	0.13
AM	V	3.15	0.75	3.61	0.78	-1.18	0.26
DM	AM	2.80	0.67	3.15	0.75	-0.98	0.34
AM	VII	3.15	0.75	3.47	0.65	-0.90	0.40
I	AM	3.47	0.97	3.15	0.75	0.73	0.47
VII	V	3.47	0.65	3.61	0.78	-0.40	0.70
I	V	3.47	0.97	3.61	0.78	-0.30	0.77
I	VII	3.47	0.97	3.47	0.65	0.02	0.98

Fermented soybean							
Group1	Group2	Group1 mean	Group1 SEM	Group2 mean	Group2 SEM	t stat	*P* value
I	AM	3.22	0.63	2.48	0.80	2.19	0.04
DM	AM	3.38	0.95	2.48	0.80	2.19	0.04
II	AM	3.19	0.65	2.48	0.80	2.07	0.05
AM	V	2.48	0.80	3.13	0.67	-1.88	0.08
DM	V	3.38	0.95	3.13	0.67	0.66	0.53
II	DM	3.19	0.65	3.38	0.95	-0.51	0.62
I	DM	3.22	0.63	3.38	0.95	-0.43	0.69
I	V	3.22	0.63	3.13	0.67	0.30	0.76
II	V	3.19	0.65	3.13	0.67	0.19	0.84
II	I	3.19	0.65	3.22	0.63	-0.11	0.91

			Equitability				
Fermented bamboo shoot							
Group1	Group2	Group1 mean	Group1 SEM	Group2 mean	Group2 SEM	t stat	*P* value
VIII	DM	0.39	0.10	0.54	0.08	-3.23	0.01
AM	VIII	0.52	0.10	0.39	0.10	2.70	0.02
VIII	VII	0.39	0.10	0.53	0.14	-2.36	0.03
VIII	II	0.39	0.10	0.49	0.11	-2.04	0.06
II	DM	0.49	0.11	0.54	0.08	-0.85	0.40
II	VII	0.49	0.11	0.53	0.14	-0.61	0.54
AM	II	0.52	0.10	0.49	0.11	0.52	0.60
AM	DM	0.52	0.10	0.54	0.08	-0.33	0.74
AM	VII	0.52	0.10	0.53	0.14	-0.19	0.86
VII	DM	0.53	0.14	0.54	0.08	-0.07	0.95
Fermented milk							
Group1	Group2	Group1 mean	Group1 SEM	Group2 mean	Group2 SEM	t stat	*P* value
II	AM	0.42	0.09	0.48	0.05	-1.69	0.11
AM	V	0.48	0.05	0.44	0.09	1.20	0.25
II	DM	0.42	0.09	0.46	0.06	-0.91	0.37
II	VII	0.42	0.09	0.46	0.09	-0.91	0.38
DM	AM	0.46	0.06	0.48	0.05	-0.80	0.44
VII	V	0.46	0.09	0.44	0.09	0.54	0.61
DM	V	0.46	0.06	0.44	0.09	0.51	0.61
AM	VII	0.48	0.05	0.46	0.09	0.58	0.64
II	V	0.42	0.09	0.44	0.09	-0.35	0.73
DM	VII	0.46	0.06	0.46	0.09	-0.06	0.96

Fermented fish							
Group1	Group2	Group1 mean	Group1 SEM	Group2 mean	Group2 SEM	t stat	*P* value
DM	V	0.53	0.09	0.64	0.10	-2.28	0.03
DM	VII	0.53	0.09	0.63	0.09	-2.20	0.04
AM	V	0.55	0.10	0.64	0.10	-1.67	0.12
I	DM	0.62	0.13	0.53	0.09	1.57	0.14
AM	VII	0.55	0.10	0.63	0.09	-1.54	0.14
I	AM	0.62	0.13	0.55	0.10	1.08	0.30
DM	AM	0.53	0.09	0.55	0.10	-0.55	0.59
I	V	0.62	0.13	0.64	0.10	-0.34	0.75
VII	V	0.63	0.09	0.64	0.10	-0.28	0.78
I	VII	0.62	0.13	0.63	0.09	-0.13	0.90
Fermented soybean							
Group1	Group2	Group1 mean	Group1 SEM	Group2 mean	Group2 SEM	t stat	*P* value
DM	AM	0.52	0.13	0.39	0.12	2.08	0.05
I	AM	0.49	0.08	0.39	0.12	1.99	0.06
II	AM	0.48	0.09	0.39	0.12	1.83	0.09
AM	V	0.39	0.12	0.48	0.09	-1.79	0.09
DM	V	0.52	0.13	0.48	0.09	0.68	0.52
II	DM	0.48	0.09	0.52	0.13	-0.63	0.55
I	DM	0.49	0.08	0.52	0.13	-0.52	0.62
I	V	0.49	0.08	0.48	0.09	0.22	0.83
II	I	0.48	0.09	0.49	0.08	-0.15	0.88
II	V	0.48	0.09	0.48	0.09	0.06	0.95

			**Coverage**				
Fermented bamboo shoot							
Group1	Group2	Group1 mean	Group1 SEM	Group2 mean	Group2 SEM	t stat	*P* value
AM	II	0.98	0.00	0.99	0.00	-3.46	0.00
II	DM	0.99	0.00	0.98	0.00	2.73	0.01
AM	VII	0.98	0.00	0.99	0.00	-2.30	0.03
AM	VIII	0.98	0.00	0.99	0.00	-2.10	0.05
VIII	II	0.99	0.00	0.99	0.00	-1.58	0.12
VII	DM	0.99	0.00	0.98	0.00	1.50	0.15
VIII	DM	0.99	0.00	0.98	0.00	1.21	0.27
AM	DM	0.98	0.00	0.98	0.00	-1.03	0.32
VIII	VII	0.99	0.00	0.99	0.00	-0.60	0.58
II	VII	0.99	0.00	0.99	0.00	0.55	0.59
Fermented milk							
Group1	Group2	Group1 mean	Group1 SEM	Group2 mean	Group2 SEM	t stat	*P* value
AM	VII	0.99	0.00	1.00	0.00	-5.81	0.00
AM	V	0.99	0.00	1.00	0.00	-4.99	0.00
II	AM	1.00	0.00	0.99	0.00	4.45	0.00
DM	VII	1.00	0.00	1.00	0.00	-3.40	0.00
DM	V	1.00	0.00	1.00	0.00	-3.10	0.01
DM	AM	1.00	0.00	0.99	0.00	3.11	0.01
II	DM	1.00	0.00	1.00	0.00	2.41	0.03
II	V	1.00	0.00	1.00	0.00	-0.91	0.38
VII	V	1.00	0.00	1.00	0.00	-0.86	0.40
II	VII	1.00	0.00	1.00	0.00	-0.17	0.86

Fermented fish							
Group1	Group2	Group1 mean	Group1 SEM	Group2 mean	Group2 SEM	t stat	*P* value
AM	V	0.98	0.00	0.99	0.00	-3.32	0.00
I	AM	0.99	0.00	0.98	0.00	3.33	0.00
AM	VII	0.98	0.00	0.99	0.00	-3.54	0.00
DM	VII	0.98	0.01	0.99	0.00	-1.52	0.15
DM	V	0.98	0.01	0.99	0.00	-1.35	0.22
I	DM	0.99	0.00	0.98	0.01	1.20	0.29
DM	AM	0.98	0.01	0.98	0.00	0.87	0.41
I	VII	0.99	0.00	0.99	0.00	-0.57	0.58
I	V	0.99	0.00	0.99	0.00	-0.36	0.74
VII	V	0.99	0.00	0.99	0.00	0.19	0.86

Fermented soybean							
Group1	Group2	Group1 mean	Group1 SEM	Group2 mean	Group2 SEM	t stat	*P* value
I	V	1.00	0.00	1.00	0.00	-1.94	0.06
I	DM	1.00	0.00	1.00	0.00	-1.64	0.11
II	V	1.00	0.00	1.00	0.00	-1.29	0.20
I	AM	1.00	0.00	1.00	0.00	-1.16	0.25
II	DM	1.00	0.00	1.00	0.00	-1.18	0.26
AM	V	1.00	0.00	1.00	0.00	-1.02	0.31
DM	AM	1.00	0.00	1.00	0.00	0.94	0.35
II	I	1.00	0.00	1.00	0.00	0.58	0.56
II	AM	1.00	0.00	1.00	0.00	-0.48	0.64
DM	V	1.00	0.00	1.00	0.00	0.25	0.80

Table S10. Additional species-level OTU extracted by the combination of three methods and the two types of mixture from four food types. For the comparsion of AM and DM with the individual extraction methods, the data obtained from each individual method were combined into a single OTU table

Fermented bamboo shoot		
II+VII+VIII	DM	AM
Acholeplasma pleciae	*Actinomyces gerencseriae*	*Acetobacter syzygii*
Acinetobacter schindleri	*Actinomyces naeslundii*	*Acetobacter tropicalis*
Acinetobacter sp. E-10	*Aeriscardovia aeriphila*	*Acholeplasma laidlawii*
actinobacterium Aac-25	*Aeromonas caviae*	*Acinetobacter lwoffii*
Actinomyces urogenitalis	*Agrobacterium vitis*	*Aeromonas hydrophila*
Anabaena azotica	*Aquaspirillum serpens*	*Agromyces luteolus*
Anaerococcus hydrogenalis	*Arthrobacter agilis*	*Amycolatopsis coloradensis*
Azospirillum amazonense	*Bacillus halmapalus*	*Arthrobacter sp. NyZ415*
Bacillus sp. 2479	*Bacillus humi*	*Asaia bogorensis*
Bacillus sp. 2-9-3	*Bacillus sp. MD2*	*Atopobacter phocae*
Bacillus sp. Bt 27	*Bacillus sp. N2a*	*Azospirillum sp. BV-s*
Bacillus sp. T03-2A	*Bacillus sp. NB-6*	*Azospirillum sp. TSH19*
Bifidobacterium longum	*Bifidobacterium thermacidophilum*	*Bacillus beijingensis*
Brevundimonas subvibrioides	*Bradyrhizobium yuanmingense*	*Bacillus catenulatus*
Brevundimonas vesicularis	*Brochothrix campestris*	*Bacillus coahuilensis*
Burkholderia sp. 383	*Brochothrix thermosphacta*	*Bacillus cohnii*
Burkholderia thailandensis	*Candidatus Koribacter versatilis*	*Bacillus cytotoxicus*
Candidatus Odyssella thessalonicensis	*Chthoniobacter flavus*	*Bacillus litoralis*
Candidatus Solibacter usitatus	*Clostridium hiranonis*	*Bacillus marisflavi*
Chlamydomonas moewusii	*Clostridium sporogenes*	*Bacillus niacini*
Clostridium longisporum	*Clostridium tyrobutyricum*	*Bacillus smithii*
Clostridium oceanicum	*Comamonas kerstersii*	*Bacillus sp. GB02-14C*
Clostridium sp. MK12	*Corynebacterium pilosum*	*Bacillus sp. KHg1*
Clostridium tertium	*Enterobacter ludwigii*	*Bacillus sp. KJW98*
Clostridium xylanolyticum	*Eubacterium biforme*	*Bacillus sp. MB-11*
Comamonas terrigena	*Fagopyrum esculentum*	*Bacillus sp. S210*
Corynebacterium mastitidis	*Frankia sp.*	*Bacillus sporothermodurans*
Deinococcus radiophilus	*Geobacillus sp. Y412MC61*	*Bradyrhizobium sp. ISLU207*
Desulfitobacterium sp. CR1	*Haemophilus influenzae*	*Brevibacillus borstelensis*
Desulfosporosinus sp. DB	*Haemophilus parahaemolyticus*	*Brevibacterium linens*
Dyella ginsengisoli	*Halomonas salina*	*Brevundimonas bullata*
Enterococcus aquimarinus	*Lactobacillus animalis*	*Brevundimonas intermedia*
Enterococcus italicus	*Lactobacillus farciminis*	*Brevundimonas sp. BAL3*
Erythrobacter sp. S14-1	*Lactobacillus paracasei*	*Butyricicoccus pullicaecorum*
Fructobacillus ficulneus	*Lactobacillus sp. 1.1424*	*Caulobacter fusiformis*
Geobacillus jurassicus	*Legionella gresilensis*	*Caulobacter sp. FWC17*
Haemophilus haemolyticus	*Listeria welshimeri*	*Clostridium baratii*
Halomonas variabilis	*Methylobacterium rhodinum*	*Clostridium paradoxum*
Holdemania filiformis	*Microbacterium hominis*	*Corynebacterium diphtheriae*
Hymenostylium recurvirostrum	*Ochrobactrum sp. mp-3*	*Corynebacterium macginleyi*
Klebsiella pneumoniae	*Oligella ureolytica*	*Corynebacterium sp. 89349*
Kluyvera ascorbata	*Paenibacillus macerans*	*Dermacoccus sp. Ellin185*
Kocuria rosea	*Paenibacillus sp. C7*	*Dietzia maris*

Lactobacillus acidifarinae
Lactobacillus equi
Lactobacillus graminis
Lactobacillus parabrevis
Leuconostoc carnosum
Leuconostoc gasicomitatum
Lysobacter sp. OC7
Macrococcus bovicus
Methylobacterium fujisawaense
Methylobacterium sp. Hojyo1
Nostoc sp. PCC 7423
Pantoea agglomerans
Paracoccus solventivorans
Pediococcus claussenii

Pediococcus ethanolidurans
Peptoniphilus sp. oral taxon 386
Phalaenopsis aphrodite
Pigmentiphaga kullae
Pisum sativum
Planococcus sp. S5
Polytoma oviforme
Propionibacterium acidipropionici
Pseudochlorella pringsheimii
Pseudomonas putida
Pseudonocardia zijingensis
Rahnella sp. 9-Pear
Rhizobium sp. LXD30
Rhodovibrio salinarum
Rhodovulum sp. CP-10
Rhodovulum sulfidophilum
Salmonella enterica
Sarcocaulon vanderietiae

Staphylococcus devriesei
Staphylococcus succinus
Stenotrophomonas rhizophila
Stichococcus bacillaris
Streptococcus anginosus
Streptococcus pluranimalium
Streptococcus pyogenes
Tissierella sp. AIP 285.00
Treponema bryantii
Trifolium repens
uncultured epsilon proteobacterium
uncultured Methylobacterium sp.
Verrucomicrobium sp. IRVE
Verticillium dahliae
Weissella viridescens
Wolbachia pipientis

Paracoccus aminophilus
Peptococcus niger
Providencia sneebia
Pseudomonas denitrificans
Pseudomonas mendocina
Pseudomonas migulae
Pseudomonas plecoglossicida
Psychrobacter arcticus
Ricinus communis

Shewanella oneidensis
Streptomyces tricolor
Streptosporangium roseum
uncultured microorganism
uncultured Serratia sp.

Yersinia pseudotuberculosis

Drimys winteri
enrichment culture bacterium
Enterococcus cecorum
Enterococcus silesiacus
Eragrostis curvula
Erwinia chrysanthemi
Escherichia coli
Exiguobacterium acetylicum
Fructobacillus pseudoficulneus

Gemella sanguinis
Granulicatella adiacens
Haemophilus sp. CCUG 15949
Hafnia alvei
Halanaerobium hydrogeniformans
Halanaerobium saccharolyticum
Halomonas aquamarina

Ignatzschineria ureiclastica
Klebsiella granulomatis
Klebsiella oxytoca
Kurthia zopfii
Lactobacillus fabifermentans
Lactobacillus manihotivorans

Lactobacillus sharpeae
Lactococcus sp. JIP 26-01
Legionella israelensis
Leptotrichia buccalis
Leptotrichia hofstadii
Leptotrichia trevisanii
Leptotrichia wadei
Leuconostoc citreum
Listeria grayi
Microbacterium arabinogalactanolyticum
Moraxella osloensis
Morganella morganii
Nocardioides sp. IC177
Oceanobacillus chironomi
Paenibacillus lentimorbus
Parvimonas micra
Phaeobacter gallaeciensis
Planomicrobium okeanokoites
Prosthecobacter debontii
Proteus vulgaris
Psychrobacter sp. J466

Rothia mucilaginosa

Rothia nasimurium
Salmonella sp.
Serratia marcescens
Sporanaerobacter acetigenes
Sporosarcina pasteurii

		Staphylococcus pseudolugdunensis
		Staphylococcus xylosus
		Streptococcus equinus
		Streptococcus minor
		Streptococcus parauberis
		Streptosporangium pseudovulgare
		Terrabacter tumescens
		Tetragenococcus solitarius
		Treponema medium
		Treponema sp.
		Treponema vincentii
		uncultured delta proteobacterium
		uncultured Ochrobactrum sp.
		Vagococcus teuberi
		Vitreoscilla stercoraria
		Weissella confusa
Fermented milk		
II+V+VII	DM	AM
Acholeplasma axanthum	*Alicyclobacillus acidoterrestris*	*Acinetobacter lwoffii*
Acidovorax valerianellae	*Anaerococcus vaginalis*	*Acinetobacter radioresistens*
Acinetobacter johnsonii	*Azospirillum irakense*	*Aquaspirillum serpens*
Aerococcus sanguinicola	*Azospirillum sp. TSH19*	*Azospirillum sp. B510*
Aerococcus urinaeequi	*Bacillus halodurans*	*Bacillus azotoformans*
Aeromonas schubertii	*Brevundimonas intermedia*	*Bacillus firmus*
Aeromonas veronii	*Chryseobacterium haifense*	*Bacillus halmapalus*
Agrobacterium larrymoorei	*Chryseobacterium soldanellicola*	*Bacillus lentus*
Aneurinibacillus thermoaerophilus	*Clostridium disporicum*	*Bacillus sp. 2-9-3*
Bacillus aquimaris	*Clostridium kluyveri*	*Bacillus sp. GB02-14C*
Bacillus caldolyticus	*Corynebacterium capitovis*	*Bacillus sp. KJW98*
Bacillus clausii	*Deinococcus proteolyticus*	*Bacillus sp. S207*
Bacillus sp. BT97	*Desulfitobacterium dichloroeliminans*	*Bacillus sporothermodurans*
Bacillus sp. GB02-31	*Enterococcus asini*	*Bradyrhizobiaceae bacterium NS28*
Bacillus sp. JAMB-204	*Fructobacillus pseudoficulneus*	*Bradyrhizobium sp. ISLU207*
Bacillus sp. KHg1	*Geobacillus thermocatenulatus*	*Brevibacterium casei*
Bavariicoccus seileri	*Haemophilus influenzae*	*Caulobacter henricii*
Bosea vestrisii	*Lactobacillus acidophilus*	*Caulobacter vibrioides*
Brevundimonas bullata	*Lactobacillus fructivorans*	*Clostridium asparagiforme*
Caloramator fervidus	*Lactobacillus graminis*	*Clostridium sp. B901-1b*
Carnobacterium gallinarum	*Lactobacillus ingluviei*	*Clostridium sp. JC3*
Carnobacterium sp. St2	*Lactobacillus mindensis*	*Clostridium thermocellum*
Citrobacter sedlakii	*Lactobacillus namurensis*	*Clostridium xylanolyticum*
Clostridium formicaceticum	*Lactobacillus pontis*	*Croomia pauciflora*
Clostridium glycolicum	*Lactobacillus sp. DCY 50T*	*Dicentra sp. Qiu 95026*
Clostridium histolyticum	*Legionella drancourtii*	*enrichment culture bacterium*
Comamonas aquatica	*Legionella feeleii*	*Enterobacter turicensis*
Corynebacterium variabile	*Leuconostoc carnosum*	*Enterococcus saccharolyticus*
Deinococcus murrayi	*Meiothermus chliarophilus*	*Eubacterium oxidoreducens*
Enterobacter ludwigii	*Pediococcus damnosus*	*Eubacterium sp. WAL 17363*

Enterobacter sp. SA-A5-114
Enterococcus hirae

Enterococcus silesiacus

Enterococcus sp. DF14

Enterocytozoon bieneusi
Erwinia amylovora
Eubacterium hallii
Frankia sp.
Hafnia alvei
Halobacillus karajensis
Ignatzschineria indica
Kluyvera ascorbata
Lactobacillus alimentarius
Lactobacillus aviarius
Lactobacillus backi
Lactobacillus crispatus
Lactobacillus equi
Lactococcus sp. JIP 26-01
Leuconostoc fallax

Listeria grayi
Macrococcus brunensis
Mannheimia sp. NCTC 11313
Mannheimia varigena
Marinobacter sp. HS7
Marinococcus halophilus
Microbacterium chocolatum
Morganella psychrotolerans
Natronobacillus azotifigens
Nesterenkonia aethiopica

Nesterenkonia halobia
Nocardioides sp. CMU5
Nocardioides sp. JS614
Oceanobacillus chironomi
Ochrobactrum grignonense
Ochrobactrum sp. mp-3
Opitutus sp. VeGlc2
Opitutus terrae
Paenibacillus lactis
Paenibacillus sp. SH-55
Pantoea agglomerans
Paralactobacillus selangorensis
Planococcus sp. L4
Prosthecobacter vanneervenii

Pseudomonas coronafaciens
Shewanella algae

Streptococcus gallolyticus

Swaminathania salitolerans

Terrabacter sp. YK3
uncultured rumen bacterium
Weissella minor
Yersinia kristensenii

Finegoldia magna
Geobacillus thermodenitrificans
Halanaerobium hydrogeniformans
Halanaerobium saccharolyticum
Lactobacillus amylophilus
Lactobacillus hammesii
Lactobacillus jensenii
Lactobacillus pentosus
Lactobacillus siliginis
Lentzea violacea
Lysinibacillus fusiformis
Megasphaera elsdenii
Methylocystis sp. Pi5/4
Nocardioides sp. AN3
Nocardioides sp. C190
Paenibacillus lentimorbus
Parvimonas micra
Pseudomonas thermotolerans
Rhodopseudomonas sp. TUT3630
Rhodovulum sulfidophilum
Ruminococcus torques
Saccharopolyspora rectivirgula
Sarcina ventriculi
Sporosarcina saromensis
Staphylococcus simulans
Thermomicrobium roseum
Tissierella sp. LBN 291
uncultured Afipia sp.
uncultured beta proteobacterium
uncultured Clostridium sp.
Virgibacillus halodenitrificans
Virgibacillus koreensis

Pseudomonas aeruginosa		
Pseudomonas pseudoalcaligenes		
Pseudomonas rhodesiae		
Pseudomonas sp. PTB2093		
Pullulanibacillus naganoensis		
Rahnella aquatilis		
Rickettsiella grylli		
Saccharopolyspora taberi		
Selenomonas ruminantium		
Shewanella sp. ANA-3		
Shewanella sp. MR-7		
Staphylococcus carnosus		
Staphylococcus massiliensis		
Staphylococcus pasteuri		
Staphylococcus pseudolugdunensis		
Staphylococcus xylosus		
Streptococcus infantarius		
Streptococcus iniae		
Streptococcus minor		
Streptococcus pluranimalium		
Streptococcus pneumoniae		
Streptococcus uberis		
Streptomyces tricolor		
Thermus igniterrae		
Trichococcus flocculiformis		
Vagococcus salmoninarum		
Virgibacillus marismortui		
Yersinia pseudotuberculosis		
Fermented fish		
I+V+VII	DM	AM
Acetobacter estunensis	*Acholeplasma brassicae*	*Acinetobacter baumannii*
Acetobacter pomorum	*Acholeplasma parvum*	*Actinomyces meyeri*
Aeromicrobium marinum	*Acinetobacter junii*	*Actinomyces naeslundii*
Alkaliphilus transvaalensis	*Bacillus litoralis*	*Actinomyces odontolyticus*
Amycolatopsis albidoflavus	*Bacillus sp. JL-26*	*Alkaliphilus oremlandii*
Aquaspirillum serpens	*Caulobacter fusiformis*	*Anaerococcus vaginalis*
Arthrobacter pascens	*Citrobacter sedlakii*	*Azolla sp. Qiu 02051*
Atopobacter phocae	*Clostridium acidurici*	*Azospirillum amazonense*
Bacillus coagulans	*Comamonas aquatica*	*Bacillus halmapalus*
Bacillus hwajinpoensis	*Corynebacterium ammoniagenes*	*Bacillus infernus*
Bacillus sp. HM06-02	*Corynebacterium casei*	*Bacillus smithii*
Bacillus sp. NB-6	*Escherichia coli*	*Bacillus sp. KJW98*
Bacillus sporothermodurans	*Eubacterium acidaminophilum*	*Bacillus sp. S210*
Bacteroides cellulosolvens	*Fluoribacter dumoffii*	*Brevibacillus agri*
Bavariicoccus seileri	*Geobacillus thermoglucosidasius*	*Chromohalobacter sp. HS2*
Bosea vestrisii	*Klebsiella sp. MNFG_801b*	*Clostridium acetobutylicum*
Brachybacterium	*Kurthia sibirica*	*Clostridium aminobutyricum*

tyrofermentans		
Bradyrhizobium japonicum	*Lactobacillus acidophilus*	*Clostridium innocuum*
Bradyrhizobium sp. ISLU207	*Lactobacillus coleohominis*	*Clostridium subterminale*
Brevibacillus laterosporus	*Lactobacillus namurensis*	*Clostridium tetani*
Brevundimonas subvibrioides	*Lactobacillus sp. DCY 50T*	*Clostridium tetanomorphum*
Campylobacter hominis	*Lactobacillus vaginalis*	*Corynebacterium callunae*
Caulobacter vibrioides	*Leuconostoc citreum*	*Dactylosporangium salmoneum*
Chlamydophila abortus	*Micrococcus lylae*	*Enterobacter sp. SA-A5-114*
Clostridium aceticum	*Micrococcus sp. SMCC ZAT351*	*Enterococcus cecorum*
Clostridium carnis	*Neisseria flavescens*	*Enterococcus durans*
Clostridium cellulolyticum	*Paenibacillus sp. KSM-N440*	*Enterococcus mundtii*
Clostridium hiranonis	*Parachlamydia acanthamoebae*	*Enterococcus sp. R-25205*
Clostridium histolyticum	*Pediococcus damnosus*	*Eubacterium ruminantium*
Clostridium limosum	*Pseudomonas aeruginosa*	*Eubacterium sp. WAL 18692*
Clostridium longisporum	*Pseudomonas alcaligenes*	*Exiguobacterium undae*
Clostridium paradoxum	*Salmonella enterica*	*Geobacillus sp. RD-2*
Clostridium saccharoperbutylacetonicum	*Staphylococcus gallinarum*	*Halanaerobium hydrogeniformans*
Clostridium sartagoforme	*Streptococcus pyogenes*	*Halanaerobium saccharolyticum*
Clostridium taeniosporum	*Terribacillus saccharophilus*	*Kluyvera ascorbata*
Clostridium thermocellum	*Trichococcus flocculiformis*	*Lactobacillus crispatus*
Clostridium xylanolyticum	*Trochodendron aralioides*	*Lactobacillus fabifermentans*
Coptotermes formosanus	*uncultured marine bacterium*	*Lactobacillus farciminis*
Corynebacterium aurimucosum	*uncultured rumen bacterium*	*Lactobacillus rhamnosus*
Deinococcus proteolyticus	*Virgibacillus sp. JM-Vb*	*Lactobacillus satsumensis*
Desulfitobacterium hafniense		*Lactobacillus similis*
Desulfosporosinus sp. DB		*Legionella feeleii*
Dietzia maris		*Listeria grayi*
Enterococcus avium		*Methylobacterium sp. DCY52*
Enterococcus gallinarum		*Natronococcus jeotgali*
Enterococcus saccharolyticus		*Nesterenkonia halobia*
Eubacterium biforme		*Nostoc sp. CENA88*
Eubacterium sp. WAL 17363		*Nostoc sp. KU001*
Frankia sp. MgI5		*Ochrobactrum grignonense*
Fructobacillus ficulneus		*Opitutus sp. VeGlc2*
Fructobacillus fructosus		*Pimelobacter simplex*
Fusobacterium ulcerans		*Planococcus sp. L4*
Fusobacterium varium		*Planomicrobium okeanokoites*
Geobacillus sp. T45		*Providencia vermicola*
Geobacillus sp. Y412MC61		*Rheinheimera sp. Chandigarh*
Hafnia alvei		*Rhizobium leguminosarum*
Halomonas aquamarina		*Serratia liquefaciens*

Kocuria rhizophila
Kribbella catacumbae
Kurthia zopfii
Kutzneria viridogrisea
Lactobacillus curvatus
Lactobacillus graminis
Lactobacillus helveticus
Lactobacillus kefiri
Lactobacillus malefermentans
Lactobacillus paracasei
Lactobacillus pentosus
Lactobacillus saerimneri
Lactobacillus vini
Legionella lytica
Leucobacter komagatae
Leuconostoc inhae
Listeria seeligeri
Mechercharimyces mesophilus
Megasphaera micronuciformis
Methylobacterium hispanicum
Methylobacterium persicinum
Mitsuokella multacida
Morganella morganii
Nocardioides sp. C190
Oxobacter pfennigii
Paenibacillus amylolyticus
Paenibacillus lactis
Paenibacillus massiliensis
Paenibacillus sp. JDR-2
Paracoccus sp. R-24652
Parvimonas micra
Peptoniphilus asaccharolyticus
Proteus vulgaris
Protochlamydia naegleriophila
Pseudomonas brenneri
Pseudomonas chlororaphis
Pseudomonas pseudoalcaligenes
Pseudomonas putida
Psychrobacter fozii
Rahnella aquatilis
Rahnella sp. 9-Pear
Raoultella terrigena
Rhizobium tropici
Spathiphyllum wallisii
Terrabacter tumescens
uncultured proteobacterium
uncultured Pseudomonas sp.
Virgibacillus marismortui

Rhodopseudomonas palustris		
rumen bacterium NK3B85		
Shewanella putrefaciens		
Shewanella sp. MR-4		
Sporanaerobacter acetigenes		
Sporosarcina saromensis		
Staphylococcus auricularis		
Staphylococcus caprae		
Staphylococcus carnosus		
Staphylococcus lugdunensis		
Staphylococcus pseudolugdunensis		
Staphylococcus vitulinus		
Streptococcus equinus		
Streptococcus pluranimalium		
Streptococcus salivarius		
Syntrophobacter wolinii		
Thauera terpenica		
uncultured delta proteobacterium		
uncultured Enterobacter sp.		
Ureibacillus thermosphaericus		
Vagococcus salmoninarum		
Veillonella ratti		
Verrucomicrobiaceae bacterium ONA9		
Vibrio fortis		
Virgibacillus carmonensis		
Weissella viridescens		
Yersinia pseudotuberculosis		
Fermented soybean		
I+II+V	DM	AM
Acetobacter pomorum	*Acinetobacter sp. E-10*	*Acholeplasma axanthum*
Acidobacterium capsulatum	*Actinomyces gerencseriae*	*Acholeplasma parvum*
Acidovorax facilis	*Actinomyces johnsonii*	*Acinetobacter lwoffii*
Acinetobacter junii	*Actinopolyspora halophila*	*Aeriscardovia aeriphila*
Acinetobacter radioresistens	*Anabaena azotica*	*Alsophila spinulosa*
Acinetobacter sp. A1PC16	*Anaerococcus hydrogenalis*	*Bacillus sp. Bt 27*
Acinetobacter sp. AD513A	*Arthrobacter oxydans*	*Bacillus sp. PL-12*
Acinetobacter xiamenensis	*Asticcacaulis biprosthecium*	*Buttiauxella agrestis*
actinobacterium Aac-25	*Azospirillum lipoferum*	*Chelatococcus asaccharovorans*
Aeribacillus pallidus	*Bacillus caldotenax*	*Chromohalobacter marismortui*
Aerococcus sanguinicola	*Bosea eneae*	*Chthoniobacter flavus*
Aerococcus urinae	*Caloramator fervidus*	*Clostridium sp. MLG480*

Aeromonas veronii
Agrobacterium vitis
Amycolatopsis coloradensis
Anoxybacillus kestanbolensis
Atopobacter phocae
Bacillus atrophaeus
Bacillus beijingensis
Bacillus caldolyticus
Bacillus catenulatus
Bacillus coahuilensis
Bacillus fastidiosus
Bacillus flexus
Bacillus halmapalus
Bacillus horikoshii
Bacillus humi
Bacillus hwajinpoensis
Bacillus indicus
Bacillus infernus
Bacillus macyae
Bacillus niacini
Bacillus sp. A49
Bacillus sp. BT97
Bacillus sp. GB02-14C
Bacillus sp. JL-39
Bacillus sp. KHg1
Bacillus sp. KHg2
Bacillus sp. KJW98
Bacillus sp. MB-11
Bacillus sp. NB-6
Bacillus sp. PS1
Bacillus sp. RH219
Bacillus sp. SG-1
Bacillus sp. T107
Bacillus vallismortis
Bifidobacterium animalis
Bifidobacterium longum
Bradyrhizobium sp. ISLU207
Bradyrhizobium sp. SEMIA 6186
Bradyrhizobium yuanmingense
Brevibacillus parabrevis
Brevibacterium mcbrellneri
Brevundimonas intermedia
Citrobacter freundii
Clostridium limosum
Clostridium sartagoforme
Corynebacterium amycolatum
Corynebacterium jeikeium
Curtobacterium albidum
Deinococcus proteolyticus
Deinococcus radiophilus
Desulfobotulus sapovorans
Desulfocella halophila
Desulfotomaculum acetoxidans enrichment culture bacterium
Enterobacter sp. SA-A5-114
Enterococcus hirae
Geobacillus caldoxylosilyticus
Haemophilus influenzae
Haemophilus sp. CCUG 15949
Halanaerobium lacurosei
Halomonas alimentaria
Intrasporangium sp. 4LS1
Kocuria rhizophila
Legionella drancourtii
Legionella fairfieldensis
Legionella feeleii
Legionella israelensis
Legionella nagasakiensis
Legionella shakespearei
Legionella sp. NML 93L054
Legionella worsleiensis
Macrococcus bovicus
Magnolia grandiflora
Marinococcus halophilus
Martelella mediterranea
Megasphaera elsdenii
Methylobacterium persicinum
Neoasaia chiangmaiensis
Nesterenkonia aethiopica
Nocardioides sp. DN36
Nostoc sp. PCC 7423
Paenibacillus massiliensis
Paenibacillus stellifer
Pelomonas saccharophila
Cronobacter muytjensii
Diplosphaera colitermitum
Eubacterium saburreum
Fusibacter paucivorans
Gemmatimonas aurantiaca
Lactobacillus aviarius
Lactobacillus kitasatonis
Legionella longbeachae
Megamonas hypermegale
Micrococcus sp. SMCC ZAT351
Nocardioides sp. SG-4G
Opitutus terrae
Ornithinibacillus californiensis
Paenibacillus sp. C7
Paracoccus solventivorans
Phegopteris hexagonoptera
Propionibacterium acidipropionici
Prosthecobacter vanneervenii
Providencia alcalifaciens
Rhizogonium paramattense
Salmonella enterica
Selenomonas lacticifex
Staphylococcus lugdunensis
Stenotrophomonas maltophilia
Streptococcus porci
Tasmannia insipida
Terrabacter sp. YK3
uncultured delta proteobacterium
uncultured Desulfobulbus sp.
uncultured Methylobacterium sp.
Verticillium dahliae

Brevundimonas subvibrioides
Brucella abortus
Brucella melitensis
Brucella suis
Caenorhabditis remanei
Caulobacter fusiformis
Chryseobacterium formosense
Clavibacter michiganensis
Clostridium cellobioparum
Clostridium formicaceticum
Clostridium longisporum
Clostridium sardiniense
Clostridium sphenoides
Comamonas testosteroni
Corynebacterium aurimucosum
Corynebacterium flavescens
Corynebacterium glutamicum
Corynebacterium simulans
Corynebacterium singulare
Daviesia angulata
Desulfosporosinus sp. DB
Draba nemorosa
Empedobacter brevis
Enterobacter aerogenes
Enterobacter amnigenus
Enterobacter asburiae
Enterobacter turicensis
Enterococcus gallinarum
Enterococcus mundtii
Enterococcus saccharolyticus
Enterococcus silesiacus
Enterococcus thailandicus
Eragrostis curvula
Erwinia papayae
Erwinia persicina
Exiguobacterium sibiricum
Geobacillus kaustophilus
Geobacillus sp. G11MC16
Geobacillus sp. Y412MC61
Gluconobacter sp. aP78
Gluconobacter thailandicus
Granulicatella adiacens
Guizotia abyssinica
Halobacillus trueperi
Halomonas aquamarina
Halomonas campaniensis
Photobacterium damselae
Prauserella rugosa
Pseudomonas japonica
Psychrobacter faecalis
Psychrobacter marincola
Pullulanibacillus naganoensis
Rahnella aquatilis
Rhodocista pekingensis
Rickettsiella melolonthae
Selenomonas noxia
Serratia rubidaea
Streptococcus infantarius
Streptococcus intermedius
Streptococcus plurextorum
Streptococcus uberis
Tepidimicrobium ferriphilum
Terrimonas ferruginea
Terrimonas lutea
Tetragenococcus solitarius
Thauera terpenica
Truepera radiovictrix
Veillonella dispar
Vibrio fortis
Virgibacillus salarius

Halomonas salina
Klebsiella pneumoniae
Klebsiella variicola
Kocuria rosea
Kutzneria viridogrisea
Lachnospira multipara
Lachnospiraceae bacterium
A4
Lactobacillus alimentarius
Lactobacillus buchneri
Lactobacillus curvatus
Lactobacillus graminis
Lactobacillus hamsteri
Lactobacillus iners
Lactobacillus
manihotivorans
Lactobacillus oris
Lactobacillus pantheris
Lactobacillus paracasei
Lactobacillus pentosus
Lactobacillus pontis
Legionella yabuuchiae
Leptolyngbya foveolarum
Leptolyngbya sp. MMG-1
Leuconostoc citreum
Leuconostoc fallax
Leuconostoc palmae
Megasphaera
micronuciformis
Meiothermus chliarophilus
Methylobacterium
hispanicum
Methylobacterium podarium
Methylobacterium populi
Methylobacterium sp.
Hojyo1
Microbacterium arabinogalactanolyticum
Microbacterium
laevaniformans
Micrococcus lylae
Nesterenkonia sp. 10004
Paenibacillus alvei
Paenibacillus barcinonensis
Paenibacillus cookii
Paenibacillus ehimensis
Paenibacillus lautus
Paenibacillus sp. HanTHS1
Paenibacillus sp. KSM-M86
Paenibacillus sp. KSM-
N440
Paenibacillus

thiaminolyticus
Pantoea agglomerans
Pantoea ananatis
Paracoccus sp. R-24652
Phaseolus vulgaris
Planomicrobium okeanokoites
Proteus penneri
Providencia rettgeri
Providencia stuartii
Providencia vermicola
Pseudomonas aeruginosa
Pseudomonas alcaligenes
Pseudomonas migulae
Pseudomonas nitroreducens
Psychrobacter aquimaris
Psychrobacter frigidicola
Psychrobacter immobilis
Rahnella sp. 9-Pear
Rhizobium leguminosarum
Rhodococcus pyridinivorans
Rothia nasimurium
Sanguibacter keddieii
Shewanella algae
Shewanella halifaxensis
Shewanella sp. ANA-3
Shewanella sp. MR-7
Shigella dysenteriae
Shigella flexneri
Siparuna decipiens
Spathiphyllum wallisii
Staphylococcus delphini
Staphylococcus devriesei
Staphylococcus gallinarum
Staphylococcus haemolyticus
Staphylococcus hyicus
Staphylococcus massiliensis
Stenotrophomonas rhizophila
Streptococcus gallolyticus
Streptococcus minor
Streptococcus suis
Streptococcus vestibularis
Streptomyces caeruleus
Strongyloides ratti
Thermincola potens
Thermoactinomyces vulgaris
Trichormus azollae
Tsukamurella pulmonis

uncultured Bacillus sp.
uncultured Bosea sp.
uncultured Klebsiella sp.
uncultured prokaryote
uncultured rumen bacterium
Veillonella parvula
Verrucomicrobiaceae bacterium CHC12
Virgibacillus carmonensis
Vitis vinifera
Weissella koreensis
Weissella viridescens
Xanthomonas campestris
Yersinia pseudotuberculosis
Zimmermannella faecalis
Zymobacter palmae

Table S11. Details of naturally fermented foods collected from Northeast India for the present study.

Food type	Substrate used	Fermentation type	Number of sample	Place of collection
Fermented bamboo shoot	Fresh, young succulent shoot sprouts of *Dendrocalamus hamiltonii* Nees & Arn. ex Munro	Solid-state fermentation, Microaerobic	2	Itanagar
			1	Ziro
			1	Jorhat
			4	Dimapur
			2	Imphal
Fermented milk	Cow (*Bos taurus*) milk	Submerged fermentation (*Yogurt*-type), Microaerobic	1	Imphal
			2	Agartala
			7	Silchar
Fermented fish	Whole dried small fish, mainly *Puntius* spp.	Solid-state fermentation, Microaerobic	3	Imphal
			2	Silchar
			2	Agartala
			2	Aizawl
			1	Shillong
Fermented soybean	Soybean [*Glycine max* (L.) Merr.] seed	Solid-state fermentation (*Natto*-type), Aerobic	2	Imphal
			2	Dimapur
			2	Aizawl
			1	Itanagar
			3	Shillong

Table S12. List of primers and PCR conditions used for PCR-DGGE and qPCR analysis.

**Primer designation**[a]	**Sequence (5′-3′)**	**Position**	**Target region**	**Target taxon**	**PCR product size**	**Molecular methods**	**Reference**	**PCR conditions**[b]
GC338f (F)[c]	ACTCCTACGGGAGGCAGCAG	364-383[d]	SSU rRNA gene V3 region	Eubacteria	237 bp[e]	PCR-DGGE and qPCR[f]	1	94 °C, 5 min; 94 °C, 60 s; 65-55 °C, 60 s (touch-down by 1 °C every 2 cycles); 72 °C, 3 min; and 94 °C, 60 s; 55 °C, 60 s; 72 °C, 3 min; 10 cycles; 72 °C, 45 min
518r (R)	ATTACCGCGGCTGCTGG	544-560[d]						
GCNL1(F)[c]	GCATATCAATAAGCGGAGGAAAAG	154-177[g]	LSU rRNA gene D1/D2 domain	Yeast	285 bp[e]	PCR-DGGE and qPCR[h]	2	95 °C, 5 min; 95 °C, 60 s; 52 °C, 45 s; 72 °C, 60 s; 30 cycles; 72 °C, 45 min
LS2 (R)	ATTCCCAAACAACTCGACTC	379-398[g]						

[a]F- proximal primer; R- distal primer.

[b]Initial denaturation, cycling denaturation, annealing and extension temperatures and durations, respectively.

[c]A 40 bp GC-clamp (5′-CGCCCGCCGCGCGCGGCGGGCGGGGCGGGGGCACGGGGGG-3′) was attached to the 5′ end of the primer.

[d]Relative position on SSU rRNA gene of *Lactobacillus plantarum* strain WCFS1 (NCBI GenBank accession no. NR_075041.1)

[e]Size includes the additional 40bp of the GC-clamp.

[f]Without the GC-clamp. The qPCR conditions are 95 °C for 5 min; cycling denaturation, annealing and extension at 95 °C for 15 s, 62 °C for 30 s and 68 °C for 45 s respectively; 40 cycles.

[g]Relative position on LSU rRNA gene of *Saccharomyces cerevisiae* (NCBI GenBank accession no. J01355.1)

[h]Without the GC-clamp. The qPCR conditions are 95 °C for 10 min; cycling denaturation, annealing and extension at 95 °C for 15 s, 52 °C for 30 s and 68 °C for 45 s respectively; 40 cycles.

References

1. Ampe F, ben Omar N, Moizan C, Wacher C, Guyot JP. (1999). Polyphasic study of the spatial distribution of microorganisms in Mexican pozol, a fermented maize dough, demonstrates the need for cultivation-independent methods to investigate traditional fermentations. *Appl and Environ Microbiol* **65:** 5464–5473.
2. Cocolin L, Aggio D, Manzano M, Cantoni C, Comi G. (2002). An application of PCR-DGGE analysis to profile the yeast populations in raw milk. *Int Dairy J* **12:** 407–411.

Table S13. List of forward and barcoded reverse primers used for amplification of eubacterial SSU rRNA gene V4-V5 region for multiplexed Illumina MiSeq sequencing. F563-577 is the proximal primer. MID1-R5 to MID100-R5 are the barcoded distal primer.

Primer type	Sequence (5' to 3')	12-bp Golay error correcting barcode
F563-577	AYTGGGYDTAAAGNG	-
MID1-R5	TCCCTTGTCTCCCCGTCAATTCMTTTRAGT	TCCCTTGTCTCC
MID2-R5	ACGAGACTGATTCCGTCAATTCMTTTRAGT	ACGAGACTGATT
MID3-R5	GCTGTACGGATTCCGTCAATTCMTTTRAGT	GCTGTACGGATT
MID4-R5	ATCACCAGGTGTCCGTCAATTCMTTTRAGT	ATCACCAGGTGT
MID5-R5	TGGTCAACGATACCGTCAATTCMTTTRAGT	TGGTCAACGATA
MID6-R5	ATCGCACAGTAACCGTCAATTCMTTTRAGT	ATCGCACAGTAA
MID7-R5	GTCGTGTAGCCTCCGTCAATTCMTTTRAGT	GTCGTGTAGCCT
MID8-R5	AGCGGAGGTTAGCCGTCAATTCMTTTRAGT	AGCGGAGGTTAG
MID9-R5	ATCCTTTGGTTCCCGTCAATTCMTTTRAGT	ATCCTTTGGTTC
MID10-R5	TACAGCGCATACCCGTCAATTCMTTTRAGT	TACAGCGCATAC
MID11-R5	ACCGGTATGTACCCGTCAATTCMTTTRAGT	ACCGGTATGTAC
MID12-R5	AATTGTGTCGGACCGTCAATTCMTTTRAGT	AATTGTGTCGGA
MID13-R5	TGCATACACTGGCCGTCAATTCMTTTRAGT	TGCATACACTGG
MID14-R5	AGTCGAACGAGGCCGTCAATTCMTTTRAGT	AGTCGAACGAGG
MID15-R5	ACCAGTGACTCACCGTCAATTCMTTTRAGT	ACCAGTGACTCA
MID16-R5	GAATACCAAGTCCCGTCAATTCMTTTRAGT	GAATACCAAGTC
MID17-R5	GTAGATCGTGTACCGTCAATTCMTTTRAGT	GTAGATCGTGTA
MID18-R5	TAACGTGTGTGCCCGTCAATTCMTTTRAGT	TAACGTGTGTGC
MID19-R5	CATTATGGCGTGCCGTCAATTCMTTTRAGT	CATTATGGCGTG
MID20-R5	CCAATACGCCTGCCGTCAATTCMTTTRAGT	CCAATACGCCTG

MID21-R5	GATCTGCGATCCCCGTCAATTCMTTTRAGT	GATCTGCGATCC
MID22-R5	CAGCTCATCAGCCCGTCAATTCMTTTRAGT	CAGCTCATCAGC
MID23-R5	CAAACAACAGCTCCGTCAATTCMTTTRAGT	CAAACAACAGCT
MID24-R5	GCAACACCATCCCCGTCAATTCMTTTRAGT	GCAACACCATCC
MID25-R5	GCGATATATCGCCCGTCAATTCMTTTRAGT	GCGATATATCGC
MID26-R5	CGAGCAATCCTACCGTCAATTCMTTTRAGT	CGAGCAATCCTA
MID27-R5	AGTCGTGCACATCCGTCAATTCMTTTRAGT	AGTCGTGCACAT
MID28-R5	GTATCTGCGCGTCCGTCAATTCMTTTRAGT	GTATCTGCGCGT
MID29-R5	CGAGGGAAAGTCCCGTCAATTCMTTTRAGT	CGAGGGAAAGTC
MID30-R5	CAAATTCGGGATCCGTCAATTCMTTTRAGT	CAAATTCGGGAT
MID31-R5	AGATTGACCAACCCGTCAATTCMTTTRAGT	AGATTGACCAAC
MID32-R5	AGTTACGAGCTACCGTCAATTCMTTTRAGT	AGTTACGAGCTA
MID33-R5	GCATATGCACTGCCGTCAATTCMTTTRAGT	GCATATGCACTG
MID34-R5	CAACTCCCGTGACCGTCAATTCMTTTRAGT	CAACTCCCGTGA
MID35-R5	TTGCGTTAGCAGCCGTCAATTCMTTTRAGT	TTGCGTTAGCAG
MID36-R5	TACGAGCCCTAACCGTCAATTCMTTTRAGT	TACGAGCCCTAA
MID37-R5	CACTACGCTAGACCGTCAATTCMTTTRAGT	CACTACGCTAGA
MID38-R5	TGCAGTCCTCGACCGTCAATTCMTTTRAGT	TGCAGTCCTCGA
MID39-R5	ACCATAGCTCCGCCGTCAATTCMTTTRAGT	ACCATAGCTCCG
MID40-R5	TCGACATCTCTTCCGTCAATTCMTTTRAGT	TCGACATCTCTT
MID41-R5	GAACACTTTGGACCGTCAATTCMTTTRAGT	GAACACTTTGGA
MID42-R5	GAGCCATCTGTACCGTCAATTCMTTTRAGT	GAGCCATCTGTA
MID43-R5	TTGGGTACACGTCCGTCAATTCMTTTRAGT	TTGGGTACACGT
MID44-R5	AAGGCGCTCCTTCCGTCAATTCMTTTRAGT	AAGGCGCTCCTT
MID45-R5	TAATACGGATCGCCGTCAATTCMTTTRAGT	TAATACGGATCG
MID46-R5	TCGGAATTAGACCCGTCAATTCMTTTRAGT	TCGGAATTAGAC

MID47-R5	TGTGAATTCGGACCGTCAATTCMTTTRAGT	TGTGAATTCGGA
MID48-R5	CATTCGTGGCGTCCGTCAATTCMTTTRAGT	CATTCGTGGCGT
MID49-R5	TACTACGTGGCCCCGTCAATTCMTTTRAGT	TACTACGTGGCC
MID50-R5	GGCCAGTTCCTACCGTCAATTCMTTTRAGT	GGCCAGTTCCTA
MID51-R5	GATGTTCGCTAGCCGTCAATTCMTTTRAGT	GATGTTCGCTAG
MID52-R5	CTATCTCCTGTCCCGTCAATTCMTTTRAGT	CTATCTCCTGTC
MID53-R5	ACTCACAGGAATCCGTCAATTCMTTTRAGT	ACTCACAGGAAT
MID54-R5	ATGATGAGCCTCCCGTCAATTCMTTTRAGT	ATGATGAGCCTC
MID55-R5	GTCGACAGAGGACCGTCAATTCMTTTRAGT	GTCGACAGAGGA
MID56-R5	TGTCGCAAATAGCCGTCAATTCMTTTRAGT	TGTCGCAAATAG
MID57-R5	CATCCCTCTACTCCGTCAATTCMTTTRAGT	CATCCCTCTACT
MID58-R5	TATACCGCTGCGCCGTCAATTCMTTTRAGT	TATACCGCTGCG
MID59-R5	AGTTGAGGCATTCCGTCAATTCMTTTRAGT	AGTTGAGGCATT
MID60-R5	ACAATAGACACCCCGTCAATTCMTTTRAGT	ACAATAGACACC
MID61-R5	CGGTCAATTGACCCGTCAATTCMTTTRAGT	CGGTCAATTGAC
MID62-R5	GTGGAGTCTCATCCGTCAATTCMTTTRAGT	GTGGAGTCTCAT
MID63-R5	GCTCGAAGATTCCCGTCAATTCMTTTRAGT	GCTCGAAGATTC
MID64-R5	AGGCTTACGTGTCCGTCAATTCMTTTRAGT	AGGCTTACGTGT
MID65-R5	TCTCTACCACTCCCGTCAATTCMTTTRAGT	TCTCTACCACTC
MID66-R5	ACTTCCAACTTCCCGTCAATTCMTTTRAGT	ACTTCCAACTTC
MID67-R5	CTCACCTAGGAACCGTCAATTCMTTTRAGT	CTCACCTAGGAA
MID68-R5	GTGTTGTCGTGCCCGTCAATTCMTTTRAGT	GTGTTGTCGTGC
MID69-R5	CCACAGATCGATCCGTCAATTCMTTTRAGT	CCACAGATCGAT
MID70-R5	TATCGACACAAGCCGTCAATTCMTTTRAGT	TATCGACACAAG
MID71-R5	GATTCCGGCTCACCGTCAATTCMTTTRAGT	GATTCCGGCTCA
MID72-R5	CGTAATTGCCGCCCGTCAATTCMTTTRAGT	CGTAATTGCCGC

MID73-R5	GGTGACTAGTTCCCGTCAATTCMTTTRAGT	GGTGACTAGTTC
MID74-R5	ATGGGTTCCGTCCCGTCAATTCMTTTRAGT	ATGGGTTCCGTC
MID75-R5	TAGGCATGCTTGCCGTCAATTCMTTTRAGT	TAGGCATGCTTG
MID76-R5	AACTAGTTCAGGCCGTCAATTCMTTTRAGT	AACTAGTTCAGG
MID77-R5	ATTCTGCCGAAGCCGTCAATTCMTTTRAGT	ATTCTGCCGAAG
MID78-R5	AGCATGTCCCGTCCGTCAATTCMTTTRAGT	AGCATGTCCCGT
MID79-R5	GTACGATATGACCCGTCAATTCMTTTRAGT	GTACGATATGAC
MID80-R5	GTGGTGGTTTCCCCGTCAATTCMTTTRAGT	GTGGTGGTTTCC
MID81-R5	TAGTATGCGCAACCGTCAATTCMTTTRAGT	TAGTATGCGCAA
MID82-R5	TGCGCTGAATGTCCGTCAATTCMTTTRAGT	TGCGCTGAATGT
MID83-R5	ATGGCTGTCAGTCCGTCAATTCMTTTRAGT	ATGGCTGTCAGT
MID84-R5	GTTCTCTTCTCGCCGTCAATTCMTTTRAGT	GTTCTCTTCTCG
MID85-R5	CGTAAGATGCCTCCGTCAATTCMTTTRAGT	CGTAAGATGCCT
MID86-R5	GCGTTCTAGCTGCCGTCAATTCMTTTRAGT	GCGTTCTAGCTG
MID87-R5	GTTGTTCTGGGACCGTCAATTCMTTTRAGT	GTTGTTCTGGGA
MID88-R5	GGACTTCCAGCTCCGTCAATTCMTTTRAGT	GGACTTCCAGCT
MID89-R5	CTCACAACCGTGCCGTCAATTCMTTTRAGT	CTCACAACCGTG
MID90-R5	CTGCTATTCCTCCCGTCAATTCMTTTRAGT	CTGCTATTCCTC
MID91-R5	ATGTCACCGCTGCCGTCAATTCMTTTRAGT	ATGTCACCGCTG
MID92-R5	TGTAACGCCGATCCGTCAATTCMTTTRAGT	TGTAACGCCGAT
MID93-R5	AGCAGAACATCTCCGTCAATTCMTTTRAGT	AGCAGAACATCT
MID94-R5	TGGAGTAGGTGGCCGTCAATTCMTTTRAGT	TGGAGTAGGTGG
MID95-R5	TTGGCTCTATTCCCGTCAATTCMTTTRAGT	TTGGCTCTATTC
MID96-R5	GATCCCACGTACCCGTCAATTCMTTTRAGT	GATCCCACGTAC
MID97-R5	TACCGCTTCTTCCCGTCAATTCMTTTRAGT	TACCGCTTCTTC
MID98-R5	TGTGCGATAACACCGTCAATTCMTTTRAGT	TGTGCGATAACA

MID99-R5	GATTATCGACGACCGTCAATTCMTTTRAGT	GATTATCGACGA
MID100-R5	GCCTAGCCCAATCCGTCAATTCMTTTRAGT	GCCTAGCCCAAT
